# The Chemistry and Biological Activities of Natural Products from Northern African Plant Families: From Taccaceae to Zygophyllaceae

**DOI:** 10.1007/s13659-016-0091-9

**Published:** 2016-03-01

**Authors:** Fidele Ntie-Kang, Leonel E. Njume, Yvette I. Malange, Stefan Günther, Wolfgang Sippl, Joseph N. Yong

**Affiliations:** Department of Pharmaceutical Chemistry, Martin-Luther University of Halle-Wittenberg, Wolfgang-Langenbeck-Str. 4, 06120 Halle (Saale), Germany; Department of Chemistry, Faculty of Science, University of Buea, P.O. Box 63, Buea, Cameroon; Department of Chemistry, Faculty of Science, Chemical and Bioactivity Information Centre, University of Buea, P.O. Box 63, Buea, Cameroon; Institute of Pharmaceutical Sciences, Research Group Pharmaceutical Bioinformatics, Albert-Ludwigs-Universität Freiburg, Hermann-Herder-Strasse 9, 79104 Freiburg, Germany

**Keywords:** African flora, Biological activities, Ethnobotany, Natural products, Traditional medicine

## Abstract

**Abstract:**

Traditional medicinal practices have a profound influence on the daily lives of people living in developing countries, particularly in Africa, since the populations cannot generally afford the cost of Western medicines. We have undertaken to investigate the correlation between the uses of plants in Traditional African medicine and the biological activities of the derived natural products, with the aim to validate the use of traditional medicine in Northern African communities. The literature is covered for the period 1959–2015 and part III of this review series focuses on plant families with names beginning with letters T to Z. The authors have focused on curating data from journals in natural products and phytomedicine. Within each journal home page, a query search based on country name was conducted. All articles “hits” were then verified, one at a time, that the species was harvested within the Northern African geographical regions. The current data partly constitutes the bases for the development of the Northern African natural compounds database. The review discusses 284 plant-based natural compounds from 34 species and 11 families. It was observed that the ethnobotanical uses of less than 40 % of the plant species surveyed correlated with the bioactivities of compounds identified.

**Graphical Abstract:**

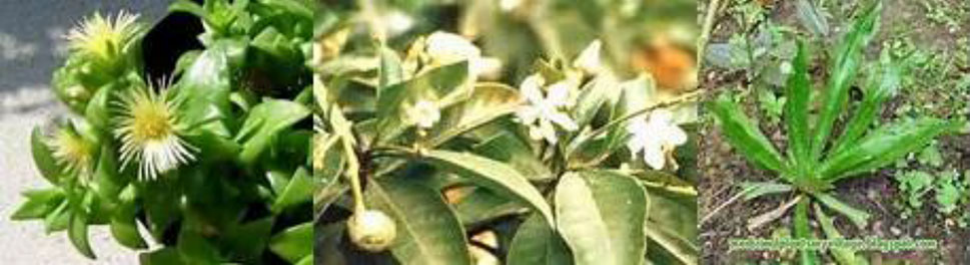

**Electronic supplementary material:**

The online version of this article (doi:10.1007/s13659-016-0091-9) contains supplementary material, which is available to authorized users.

## Introduction

Northern Africa includes the countries Algeria, Egypt, Libya, Morocco, North Sudan, South Sudan, Tunisia and Western Sahara, covering a land surface of about 8935659 km^2^ [1]. This region is mostly covered by the Sahara desert (a surface area of >50 % of the total area of the region), the rest of the region being oases and grasslands, with a total of 343 vascular plant species from 69 families non-native to this region [2]. The use of these plants for the treatment of disease ailments is known and documented from the Ancient Egyptian civilization [3]. Till today, diverse plant parts (roots, rhizomes, flowers, leaves, fruits, seeds and oils; in the form of powders, pills, suppositories, creams, pastes, and ointments, or sometimes combinations of these) are often used locally in traditional medicine within this region and the herbal material is commonly commercialized in the local markets. Urbanization and desertification however represent constant threats to some of the plant species [4–6]. Although Arabic traditional medicine is in danger of disappearing as a result of the influence of Western medicine [7], the use of plant-based medicines in Africa, as a whole, dates back to several centuries and several attempts have been made by government authorities to ensure its continuity [8]. Additionally, scientists have attempted to record and study the old recipes with the view of identifying the active principles in the herbal preparations and understanding the modes of action of the bioactive principles, with the ultimate goal of developing phytomedicines and/or drugs from these preparations [6]. It should be noted that traditional medicine is not just commonly practised in Northern Africa, but in the entire continent, to the extent that traditional medicine has been included as one of the hubs of the African Network for Drugs and Diagnostics Innovation (ANDI) [9–11].

The assertion that natural products represent a huge potential for drugs and drug leads cannot be disputed [12]. This is because nature uses exquisite enzyme reaction sequences, conducts many chemical transformations with high regio- and stereospecificity, leading to the formation of secondary metabolites [13]. These secondary metabolites (originally designed by plants for their own defence mechanism and survival) could be used directly as drugs or further chemically modified to give drug molecules. A quick search of the literature (the main stream natural products journals and PhD theses from university libraries in Northern Africa) could give an estimate of >3000 unique compounds which have been previously isolated from Northern African flora, algae and fauna. Although the database of natural compounds from Northern African sources is still under construction, a recent survey has been undertaken by two of us to investigate the correlation that exists between the biological activities of the isolated metabolites from Northern African floral matter and the use of the respective plant species in local traditional medicine [14, 15]. The current review paper represents a continuation of this survey, with plants belonging to families having names beginning with letters T to Z (34 species and 11 families). As in the two previous reviews, the plant families have been presented in alphabetical order. Wherever the biological activities of the isolated metabolites correlate with the ethnobotanical uses of the plant species, these are highlighted in bold in the Tables.

## Taccaceae and Tamaricaceae

Taccaceae or Dioscoreales (the family of yams), particularly the genus *Tacca*, is mostly characterised by the presence of taccalonolides (a new class of plant-derived natural steroids with a microtubule-stabilizing activity, withanolides and their glucosides, saponins, pregnane glycosides, diarylheptanoids and their glycosides, the isolated compounds mostly showing cytotoxic, microtubule-stabilizing, NF-κB activation, PPAR transcriptional and insecticidal activities [16]. A summary of the medicinal uses and biological activities of the compounds of the Taccaceae and Tamaricaceae from Northern Africa are shown in Table 1. The plant species *Tacca leontopetaloides* (Taccaceae or Dioscoreaceae, the yam family) is native to tropical Africa, South Asia, Southeast Asia, Northern Australia, New Guinea, Samoa, Micronesia, and Fiji [17]. The species plays important roles in the environment (ornamental), as a human food source and in folklore medicine. The roots are known to contain a number of potent molluscicidal steroidal saponins [18], while the bitter principle taccalin (**1**), along with an ester and alcohols have also been isolated from the plant [19]. From the leaves of the plant harvested from Southern Sudan, Abdel-Aziz et al. successfully isolated the B-ring contracted spirotane, leontogenin (**2**) for the very first time [20]. This plant is also known to contain the microtubule stabilizers; taccalonolides A, B, E and N (**3**–**6**), which have clinical potential for the treatment of cancer [21]. Jagtap and Satpute have recently identified flavonoids (like diosmin, rutin, epigenin, hesperidin, quercetin and isoquercetin by HPTLC) from the tubers of this plant and have attributed the presence of these flavonoids to the food value of the plant tubers [22]. While the presence of the cytotoxic components (**3**–**6**) could explain the poisonous nature of the tubers of this plant, additional uses in folklore medicine are described in the database of plants from Micronesia [23].
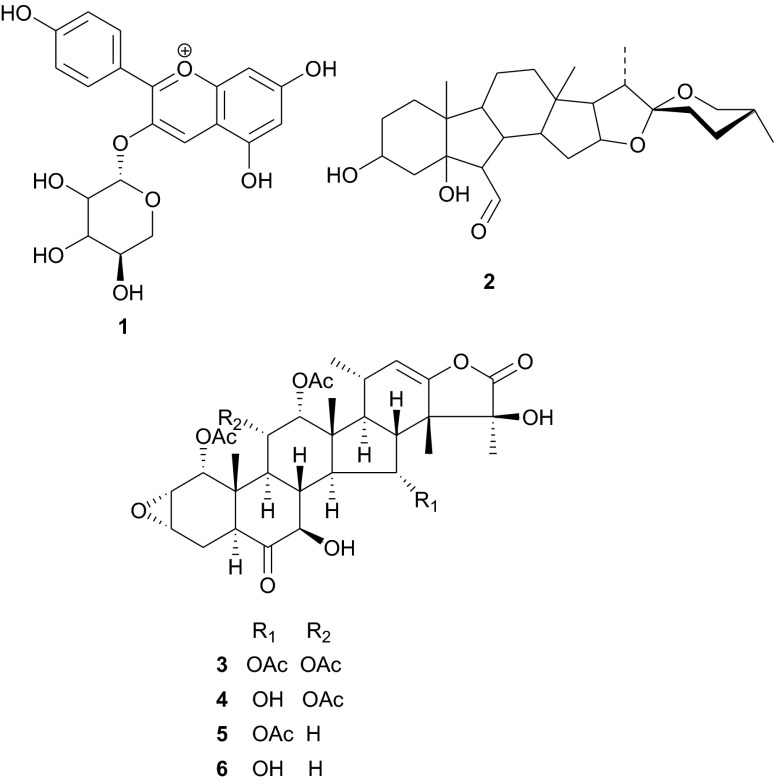

**Table 1:** Summary of ethnobotanical uses versus measured biological activities of isolated secondary metabolites from Taccaceae and Tamaricaceae

Plant family	Plant name (country)	Use in traditional medicine	Part of plant studied	Isolated principle	Measured activity	Author and reference
Taccaceae or dioscoreaceae	*Tacca leontopetaloides* (Sudan)	Diverse uses, including; the tuber serves as an important food and the starch is used to stiffen fabrics. The bitter raw tubers are generally used to treat stomach ailments (mainly diarrhea and dysentery)	Leaves	**1**–**6**	Microtubule stabilizing properties	Abdel-Aziz et al. [20]; Risinger and Mooberry [21]
Tamaricaceae	*Tamarix aphylla* (Egypt)	Shade tree and fire barrier	Stem bark	**9**–**12**	Not tested	Souliman et al. [24]; Merfort et al. [25]
			Leaves	**13**–**21**	Significant human tumour-selective cytotoxic activities	Orabi et al. [27]
			Galls	**22**–**33**	Not tested	Orabi et al. [28]; Nawwar et al. [30]
	*Tamarix nilotica* (Egypt)	Used in the Egyptian traditional medicine as an antiseptic agent. The plant has been used to expel fever, relieve headache, to draw out inflammation, and as an aphrodisiac. The flowers have demonstrated hepatoprotective and **antioxidant** activities	Flowers	**34**–**36**	Not tested	Nawwar et al. [33]; Nawwar and Souleman [34].
			Leaves	**37**–**62**	**39**,** 40** and** 42** showed **1,1-diphenyl-2-picrylhydrazyl radical scavenging activity**, while **45** and **57** showed cytotoxic effects	Abouzid et al. [36]; Orabi et al. [37, 38]
			Roots	**63**–**70**	Not tested	Barakat et al. [39]; Nawwar et al. [40]
	*Reaumuria vermiculata* (Egypt)	The plant decoction is used externally or taken internally to cure fromitch and bruises [50]	Aerial parts	**82**–**86**	Antioxidant and cytotoxic activities	Nawwar et al. [49]

The Tamaricaceae (the tamarisk family) are mostly composed of phenolics and gall polyphenolics (flavonoids, tannins, phenolic aldehydes), terpenoids, ellagic acid derivatives and ferulic acid derivatives. The most investigated species of the Tamaricaceae from North Africa are of the *Tamarix* genus (*T. aphylla* and *T. nilotica*) [24–40]. Several metabolites have been isolated from *T. aphylla*, including the unique diaryloxy furanofuran lignan, (+)-2_e_,6_e_-bis-(1-oxy-2,3-dimethoxyphenyl)-3,7-dioxabicyclo-[3,3,0]octane (**7**), the polyphenolic l′-decarboxydehydrodigallic acid (**8**) and the glyceryl ester, 1-isoferulyl-3-pentacosanoyl glycerol (**9**) from the stem bark [24]. From the stem bark of the same Egyptian species, Merfort et al. further isolated the triterpenes; isomyricadiol (**10**), its 3β isomer myricadiol (**11**), along with its 3-ketone (**12**) [25]. From the leaves, Saleh et al. identified the sulphunated flavonoid, rhamnetin glucuronide trisulphate [26], while Orabi et al. recently isolated the cytotoxic tannins, including three new ellagitannin monomers, nilotinins M5–M7 (**13**–**15**), a dimer, nilotinin D10 (**16**), and a trimer, nilotinin T1 (**17**), together with three known dimers, hirtellin D (**18**) and tamarixinins B (**19**) and C (**20**), and a trimer, hirtellin T2 (**21**) as the major constituents of the MeOH and acetone extracts [27]. From the galls of this plant, Orabi et al. had also previously isolated an ellagitannin, phyllagallin M1 (**22**), a gallo-ellagitannin, phyllagallin D1 (**23**), and four gallotannins, phyllagallin M2 (**24**) and phyllagallins D2–D4 (**25**–**27**), in addition to known ellagitannins and three phenolics of lower molecular weight, structurally related to hydrolyzable tannins [i.e. 3,3′-di-*O*-methylellagic acid-4′-*O*-β-d-glucopyranoside (**28**), dehydrotrigallic acid (**29**), and flavogallonic acid dilactone (**30**)] [28]. Meanwhile, Nawwar et al. had isolated the galloyl glucoses; 2,6-digalloyl glucose and 3,6-digalloyl glucose [29], and the novel natural polyphenol, 2-*O*-galloyl-3-*O*-(3,4,5,6,7-pentahydroxybiphenyl ether-8_a_-carboxylic acid-1-carboxyloyl)-4,6-(*S*)-hexahydroxybiphenoyl-(α/β)-^4^C_1_-glucopyranose, commonly named tamarixellagic acid (**31**), together with the known dehydrodigallic and dehydrotrigallic acids (**32** and **33**) [30]. El Ansari et al. had also isolated the sulphunated and acylated flavonols; kaempferol-7,4′-dimethyl-ether-3-sulphate and quercetin 3-*O*-isoferulyl-β-glucuronide from the flowers of this plant [31]. Traditionally, this plant has been used as a shade tree in horticulture and locally as a fire barrier, since the high salt content of its leaves makes it difficult to burn [32]. The tested biological activities of the isolates rather show that compounds **17**, **19** and **20** have significant human tumour-selective cytotoxicities which were higher than those of synthetic and natural potent cytotoxic compounds, including polyphenols, and comparable with those of 5-fluorouracil and melphalan [27].

The investigation of the sister species *T. nilotica* yielded several metabolites including, from the flowers, the digalloylglucose nilocitin (**34**), which was the first example of a galloyl glucose not substituted at the anomeric position [33], along with phenolic lactone 3,4,8,9,10-pentahydroxy-dibenzo[*b*,*d*]pyran-6-one (**35**) and ellagic acid (**36**) [34]. Nawwar et al. also isolated flavonoids from the flowers of this species, viz, the ethyl ester of kaempferol 3-*O*-β-d-glucuronide, the methyl and ethyl esters of quercetin 3-*O*-β-d-glucuronide, together with kaempferol 3-*O*-sulphate-7,4′-dimethyl ether and the free aglycones from the aqueous extract [35]. From the leaf extracts Abouzid et al. isolated methyl ferulate 3-*O*-sulphate (**37**) for the first time from a natural source, together with coniferyl alcohol 4-*O*-sulphate (**38**), kaempferol 4′-methyl ether (**39**), tamarixetin (**40**) and quercetin 3-*O*-β-d-glucupyranuronide (**41**) from the *n*-butanol soluble fraction of the extract and pentacyclic triterpenoid, 3α-(3″,4″-dihydroxy-*trans*-cinnamoyloxy)-d-friedoolean-14-en-28-oic acid (**42**) from the *n*-hexane soluble fraction of the extract [36]. Hydrolyzable tannins were also isolated by Orabi et al. from the aqueous acetone extract of *T*. *nilotica* leaves, particularly three new hellinoyl-type ellagitannins; nilotinins M4 (**43**), D7 (**44**), and D8 (**45**), and a new macrocyclic-type, nilotinin D9 (**46**), along with eight known tannins, hirtellins C (**47**), and F (**48**), isohirtellin C (**49**), tellimagrandins I and II, and 1,2,6-tri-*O*-galloyl-β-d-glucose (**50**), hirtellin B (**51**) and tamarixinin A (**52**) [37], as well as the novel ellagitannin monomer, nilotinin M1 (**53**), and three dimers, nilotinins D1 (**54**), D2 (**55**) and D3 (**56**), together with six known tannins, the dimer hirtellin A (**57**), remurins A (**58**), and B (**59**), 1,3-di-*O*-galloyl-4,6-*O*-(*S*)-hexahydroxydiphenoyl β-d-glucose (**60**), gemin D (**61**) and hippomanin A (**62**), the last two being both monomers which were isolated for the first time from this plant species [38]. From the roots of the plant, Barakat et al. isolated the novel glyceride, niloticol, the novel aldehyde, isoferulaldehyde (**63**), together with the known aldehyde, ferulaldehyde (**64**) [39]. Meanwhile, Nawwar et al. had earlier reported the isolation of the furanofuran lignan (±)-syringaresinol (**65**), reported for the first time from the Tamaricaceae, together with the new natural product ellagic acid 3,3′-dimethyl ether 4-*O*-β-d-glucopyranoside (**66**), in addition to the known constituents; isoferulic acid (**67**), gallic acid (**68**), dehydrodigallic acid (**69**) and an ellagic acid derivative (**70**) [40]. An examination of the aerial parts of the species harvested in Saudi Arabia rather yielded several polyphenolic constituents, including the new pentacyclic triterpenoid, 3-*O*-*trans*-caffeoylisomyrica-diol (**71**), in addition to ten known compounds viz *N*-*trans*-feruloyltyramine (**72**), β-sitosterol (**73**), clematine (**74**), isoferulic acid methyl ester (**75**), ellagic acid (**76**), its 3-methyl ether (**77**), naringenin (**78**), kaempferol-7,4′-dimethyl ether (rhamnocitrin-4′-methyl ether) (**79**), kaempferide (**80**) and dilleneti (**81**) [41].
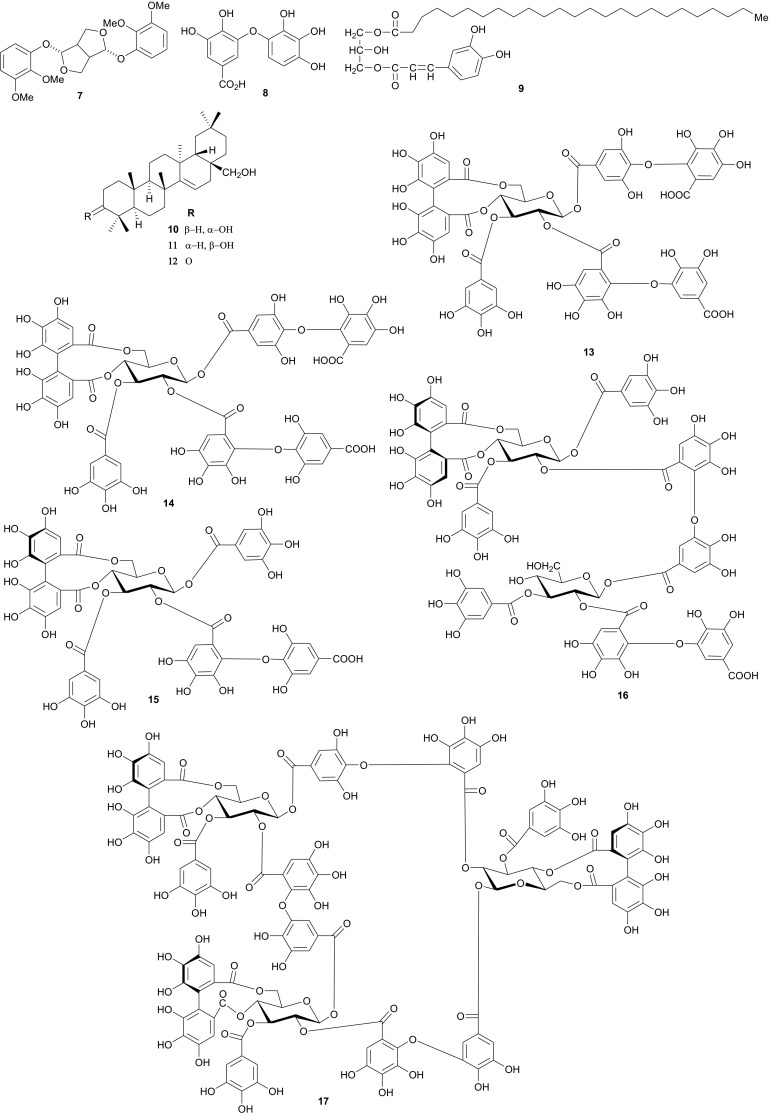

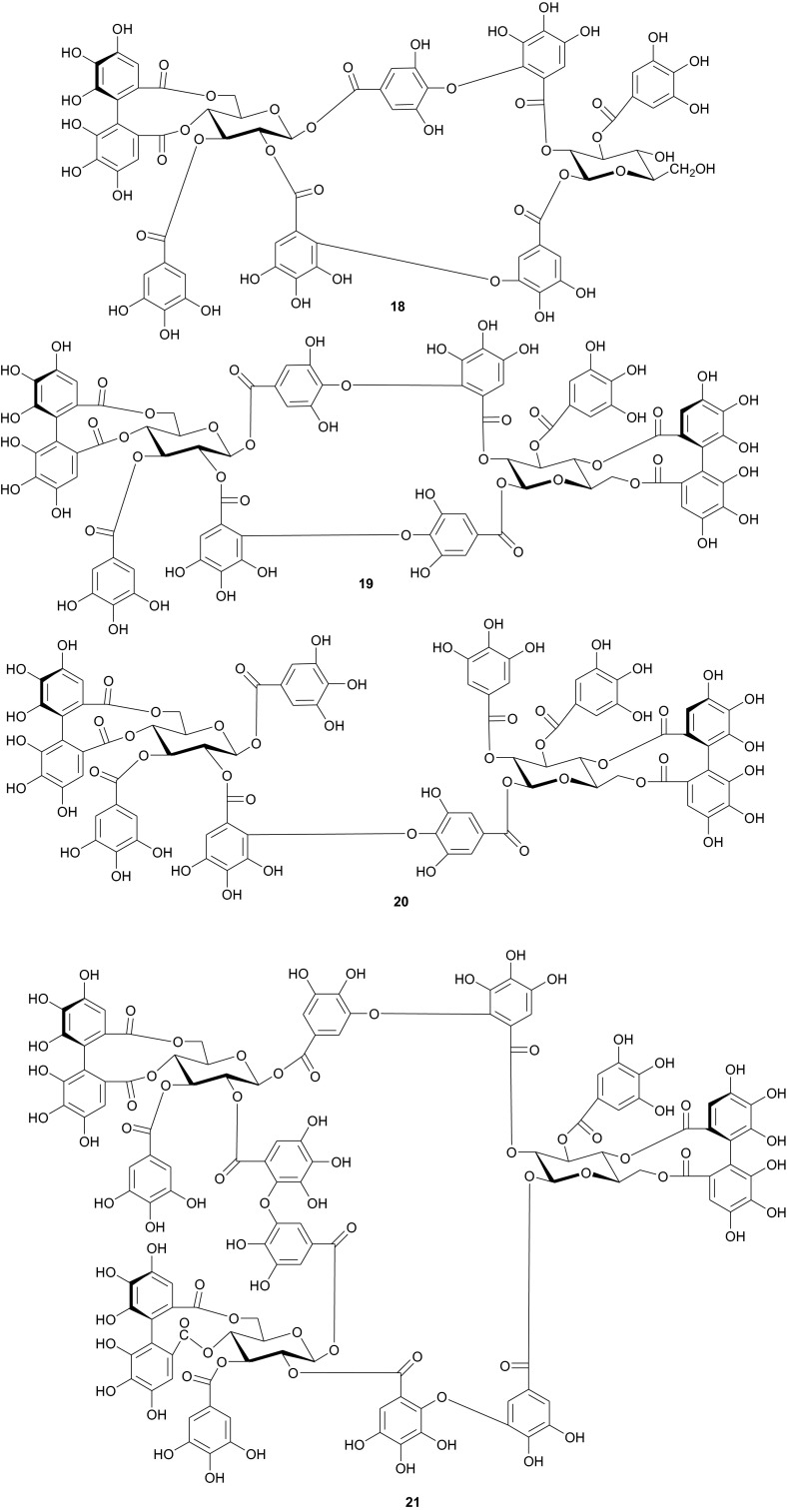

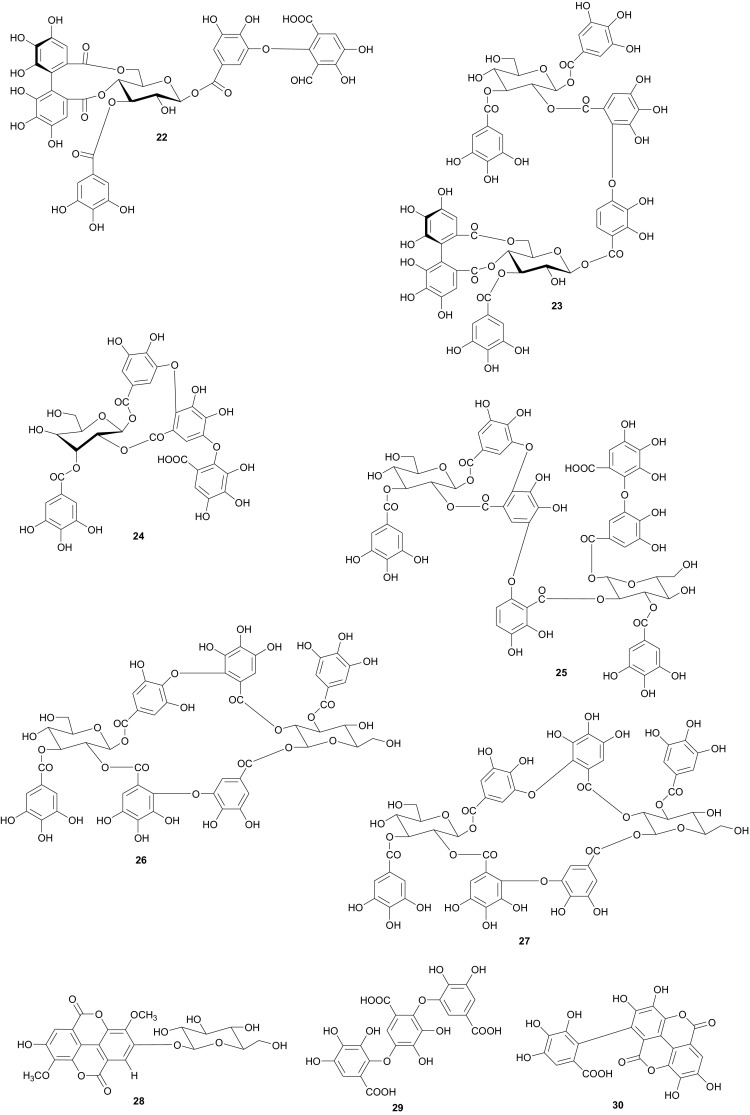

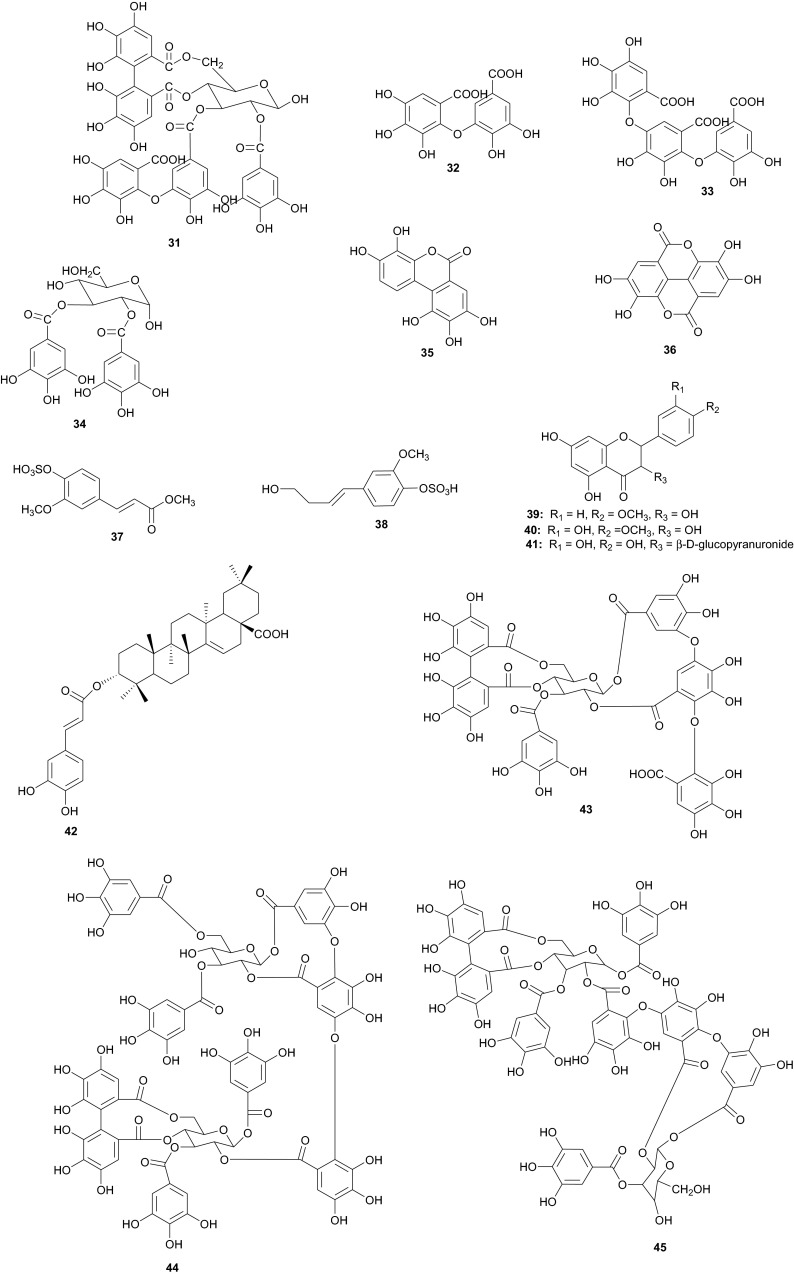

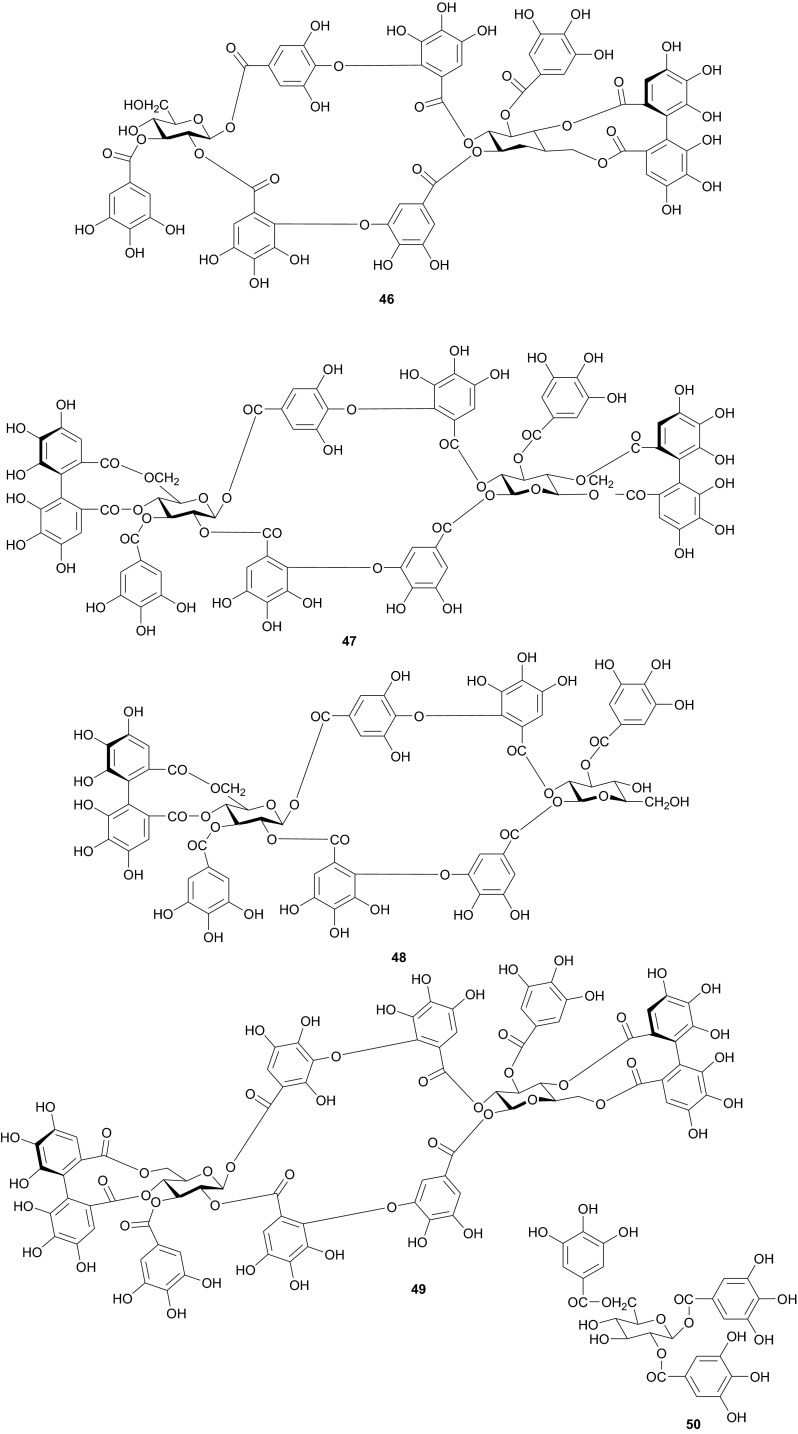

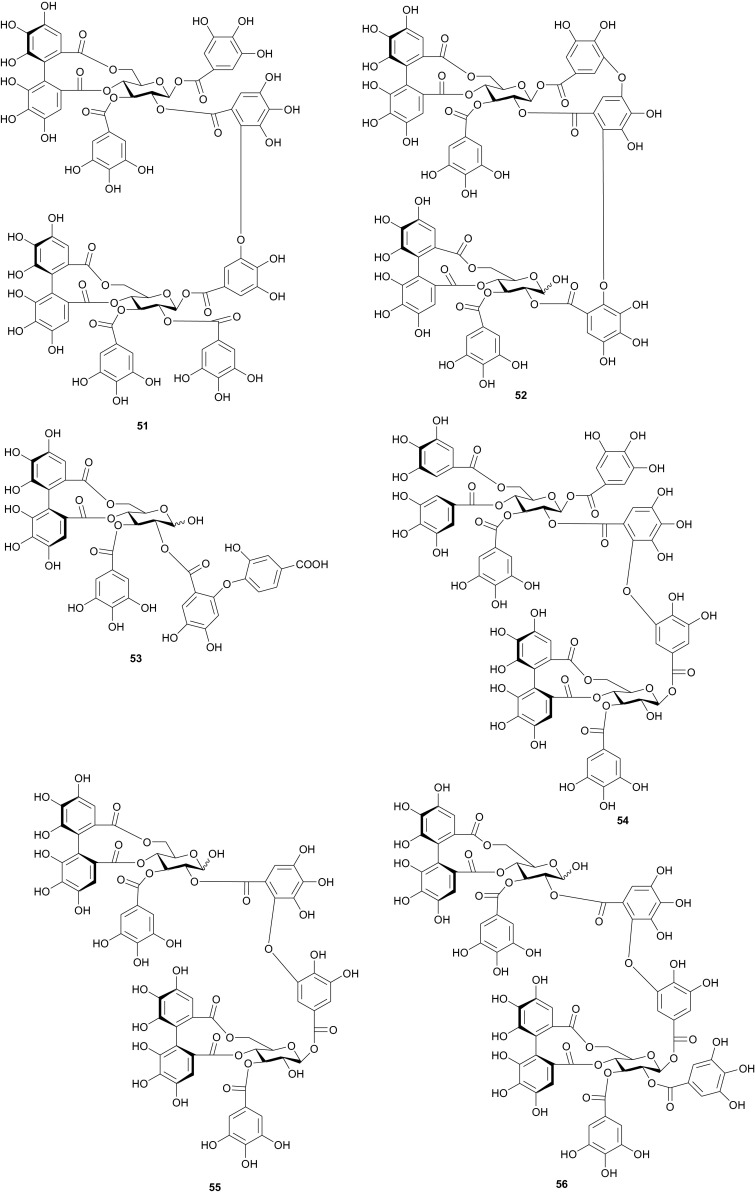

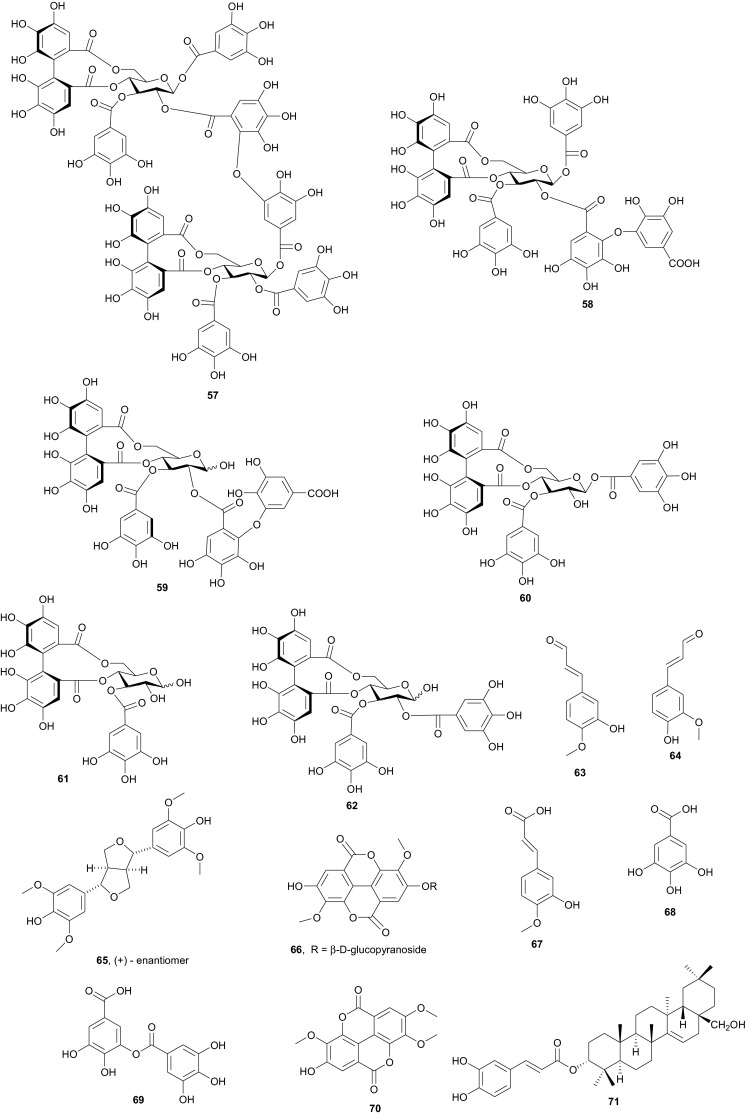

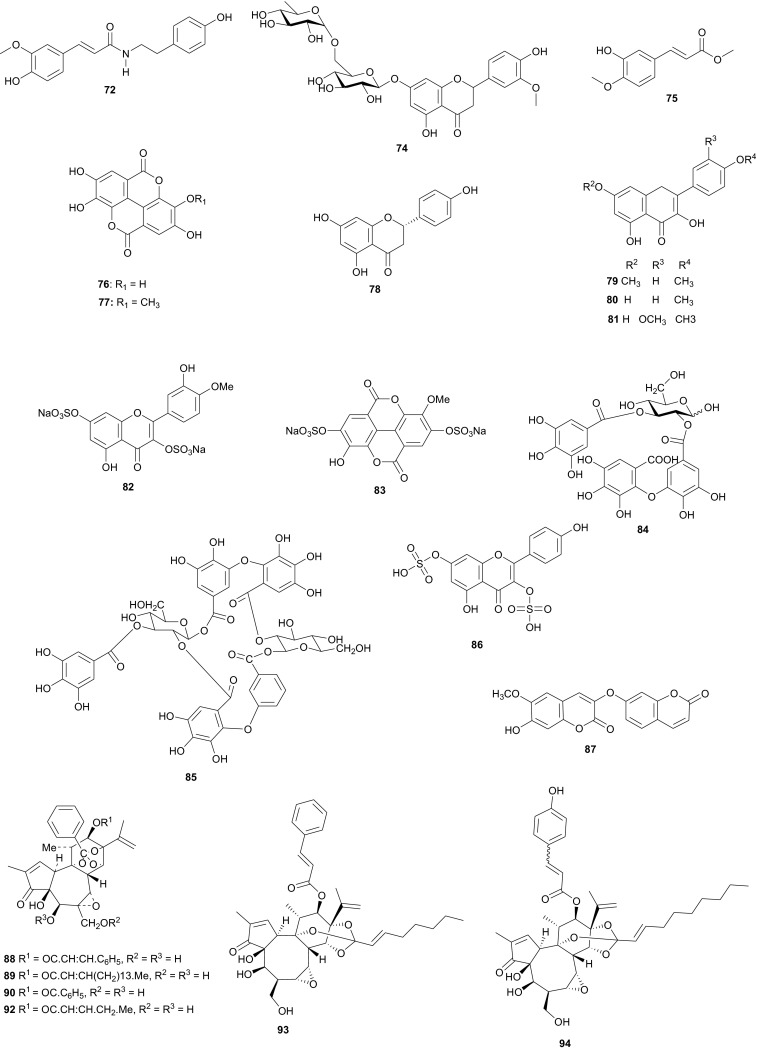


The biological activities of the tested isolated principles shows that compounds **39**, **40** and **42** exhibited 1,1-diphenyl-2-picrylhydrazyl radical scavenging activity with IC_50_ values of 35.2, 37.0 and 21.2 µM, respectively [36]. Meanwhile, the hydrolyzable tannins from the leaves showed cytotoxic activity against four human tumor cell lines at the lower micromolar range; compounds **45** and **57** showing the highest cytotoxic effects, with compound **45** exhibiting CC_50_ values of 22.9 and 32.3 µM against promyelocytic leukemia (HL-60) and human squamous carcinoma (HSC-2) cell lines respectively, while compound **57** showed respective values of 18.4 and 31.3 µM [37]. Of the compounds isolated from the Saudi species, compound **71** demonstrated better antioxidant activity (IC_50_ value of 3.56 µM) than the reference compound quecertin (5.72 µM) [41], apart from the multiple known biological activities of the other isolates like β-sitosterol (e.g. antioxidant [42], anticancer [43], analgesic [44] and anti-inflammatory [44, 45]), ellagic acid (e.g. anti-proliferative [46], antioxidant and anticancer [47, 48]), just to mention these two compounds.

Nawwar et al. have also recently isolated twenty phenolic compounds from the aerial parts of *Reaumuria vermiculata* (Tamaricaceae) [49], a plant whose decoction is used externally or taken internally to cure fromitch and bruises [50]. The isolates included four metabolites which had not been reported previously to occur as natural products, namely; tamarixetin 3,7-disulphate (**82**), 3-methoxyellagic acid 4,4′-disulphate (**83**), 2-*O*-dehydrodigallic acid monocarboxyloyl-3-*O*-galloyl-(α/β)-glucose (**84**) and the ellagitannin dimer, vermaculitin (**85**) [49, 50], meanwhile kaempferol-3,7-disulphate (**86**) is known to have been isolated for the first time from the Northern African species [51]. Compounds **84** and **85** were shown to be cytotoxic ellagitannins, which demonstrated radical scavenging capacity using both the DPPH method and the ORAC assay. Additionally, both compounds were cytotoxic against prostate cancer cell lines (PC-3), with IC_50_ values of 1.5 and 0.45 µM respectively [49, 50]. Plants of the Tamaricaceae, particularly *T. nilotica*, have been investigated for their anticancer properties [52]. It is also recorded in ancient Egyptian papyri that this plant was used to expel fever, relieve headache, draw out inflammation and as an aphrodisiac, in addition to its use in Egyptian traditional medicine as an antiseptic agent [53]. In Egypt, different parts of *Tamarix* are used in traditional medicine. For example, the leaves and young branches are cooked for oedema of spleen and mixed with ginger for uterine infections, while the bark, when boiled in water with vinegar is used as lotion against lice [54]. The above uses clearly correlate with some of the aforementioned biological activities of the isolates.

## Thymelaeaceae and Tribulaceae

The Thymelaeaceae are known for the presence of daphnane diterpenes, coumarins and a broad range of other compounds, including lignans and phenolics. A summary of the medicinal uses and biological activities of the compounds of the Northern African Thymelaeaceae and Tribulaceae are shown in Table 2. From the Thymelaeaceae, the most investigated species from Northern Africa are those of the genus *Thymelaea* (*T. hirsuta* and *T. lythroides*, the latter being more common in Morocco, while the former is the only species known in Egypt). The coumarin daphnoretin (**87**) was isolated from the leaves [55] and roots [56] of *T. hirsuta*, the 12-hydroxy-daphnane esters; gnidicin (**88**), gniditrin (**89**) and genkwadaphnin (**90**), the aliphatic C-12 ester, 12-*O*-heptadecenoyl-5-hydroxy-6,7-epoxy-resiniferonol-9,13,14-orthobenzoate (**91**) and the novel aliphatic C-12 ester 12-*O*-butenyl-5-hydroxy-6,7-epoxy-resiniferonol-9,13-14-orthobenzoate (**92**) were isolated from the leaves and twigs [57] while the daphnane diterpenoids; hirseins A (**93**) and B (**94**) were isolated from the aerial parts [58], and the flavonoid vicenin-2 (**95**) was isolated from the leaves [51].
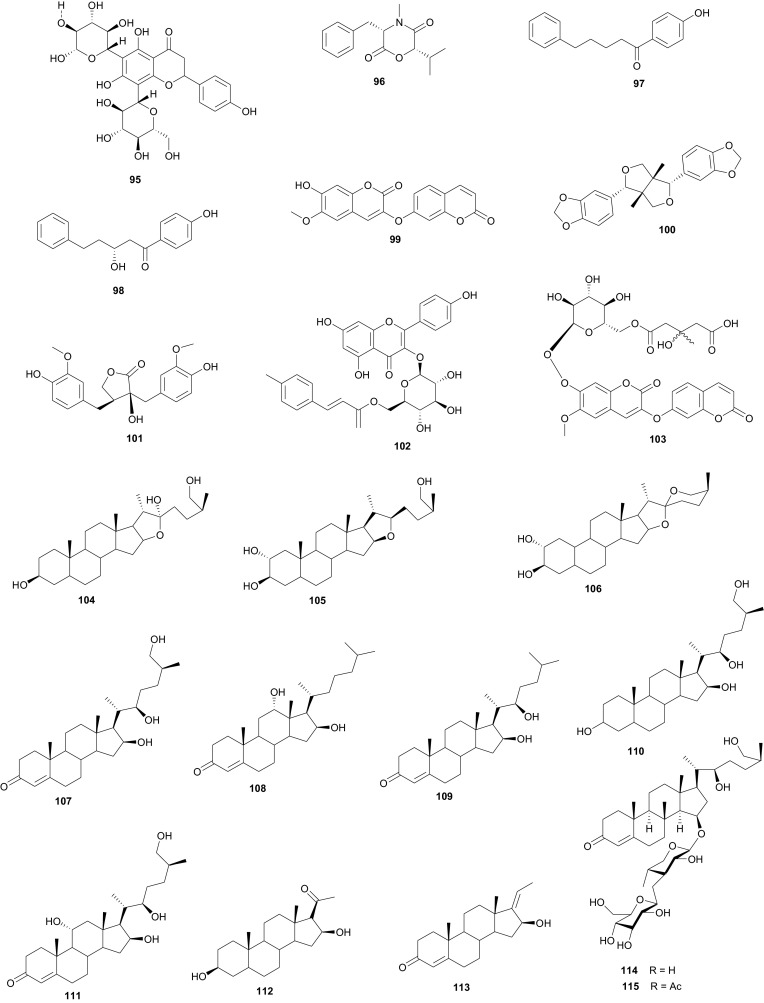

**Table 2:** Summary of ethnobotanical uses versus measured biological activities of isolated secondary metabolites from Thymelaeaceae and Tribulaceae

Plant family	Plant name (country)	Use in traditional medicine	Part of plant studied	Isolated principle	Measured activity	Author and reference
Thymelaeaceae	*Thymelaea hirsuta* (Egypt)	Used traditionally as an antiseptic and anti-inflammatory agent and for the treatment of hypertension by external application [58]. Generally, *Thymelaea* species are used for the treatment of **uterine cancer, prostate inflammation** and other related infirmities	Leaves and roots	**87**	Known **cancer chemopreventive agent** and **antiproliferative agent**	Rizk and Rimpler [55]; Abou-Karam et al. [56]
			Leaves and twigs	**88**–**92**	Not tested	Brooks et al. [57]
			Aerial parts	**93**–**94**	Inhibition of melanogenesis in B16 murine melanoma cells	Miyamae et al. [58]
			Leaves	**95**	Not tested	Nawwar et al. [51]
	*Thymelaea lythroides* (Morocco)	Used to treat a wide range of diseases, including; prostate inflammation, diabetes, rheumatism, otitis and cancer of the uterus	Aerial parts	**96**–**103**	Not tested	Kabbaj et al. [59]
Tribulaceae	*Tribulus pentandrus* (Egypt)	Plants from this genus have diverse uses, e.g. *T. terrestris* is used increase appetite, increase sexual desire in humans, regulate heart rate, blood pressure and cholesterol levels, treatment of prostatic hyperplasia, reducing the symptoms and prostate volume, etc [75]	Aerial parts	**114**–**120**	Compound **114** showed antimicrobial activities, but compounds **115–120** were not tested	Mahalel [72]; Hamed et al. [73]

From the aerial parts of *T. lythroides*, Kabbaj et al. successfully isolated eight secondary metabolites, including the depsipeptide bassiatin (**96**); the coumarins daphenone (**97**) and daphnelone (**98**), the dicoumarin daphnoretin (**99**), the lignans; δ-sesamin (**100**) and wikstromol (**101**), the flavonoid glucoside *trans*-tiliroside (**102**), and the dicoumarin rutarensin (**103**) [59]. Although the isolated compounds were not tested by the authors of the aforementioned paper, it was suggested that δ-sesamin (**100**), wikstromol (**101**) and *trans*-tiliroside (**102**) might be considered as important chemotaxonomic markers of the species [59], a claim which is still under investigation.

Among the isolated compounds, daphnoretin (**87**) is known to be a cancer chemopreventive agent by inhibiting tyrosine-specific protein kinase, with insignificant cytotoxicity against human cell lines [56], while the hirseins (**93** and **94**) were shown to be inhibitors of melanogenesis in B16 murine melanoma cells [58]. Some of the 12-hydroxy-daphnane esters are known to activate protein kinase C (the tumour-promoting receptor site) [60] and are further claimed to possess anti-tumour activities in vivo against P-388 leukemia in mice [61, 62]. Furthermore, the mechanism of antiproliferative activity of daphnoretin has been recently explored, showing that the compound causes death of human osteosarcoma (HOS) by blocking cells successively in G2/M phases and activating the caspase-3 pathway [63]. It has recently been shown that this compound displays antiviral activity [64], induces respiratory burst in rat neutrophils through protein kinase C activation [65, 66] and is a well known antineoplastic agent (inhibits or prevents the proliferation of neoplasms) [67].

Plants from the genus *Thymelaea* are known to be used to treat a wide range of diseases, including; prostate inflammation, diabetes, rheumatism, otitis and cancer of the uterus (particularly *T. lythroides*) [68]. This has been confirmed by a recent ethnopharmacological survey of traditional plants used for cancer treatment in Morocco [69]. In Tunisia, *T. hirsuta* is used traditionally as an antiseptic and anti-inflammatory agent and for the treatment of hypertension by external application [58]. The anticancer properties of the aforementioned compounds could justify the use of *Thymelaea* species for the treatment of uterine cancer, prostate inflammation and other related infirmities.

Meanwhile, the Tribulaceae are known for steroids (steroidal saponins and steroidal glycosides). From the Northern African Tribulaceae, the remarkable species investigated are of the genus *Tribulus* (*T. pentandrus*, *T. terrestris, T. megistopterus* subsp. *pterocarpus* and *T. parvispinus*), harvested in Egypt [70–73]. Traditionally, *T. terrestris* is known for diverse uses [74], including the ability to increase appetite, increase sexual desire in humans, regulate heart rate, blood pressure and cholesterol levels, treatment of prostatic hyperplasia, reducing the symptoms and prostate volume, etc. [75], while the organic and aqueous extracts from the fruits, leaves and roots have shown antimicrobial properties [76]. The plant is also claimed to possess anticancer [77], anticholinergic [78], antifilarial [79], anti-malarial [80], CNS depressant and stimulant [81], hypoglycemic effect [82], immunologic effect [83], smooth muscle relaxant and stimulant activity [84] and other important activities [85]. Kostova and Dinchev have previously published a review on the chemical diversity of saponins isolated from this species [86]. From the Northern African species from this genus, Saleh et al. detected twenty five flavonoid glycosides from extracts of *T. pentandrus* and *T. terrestris,* belonging to the common flavonols; kaempferol, quercetin and isorhamnetin, together with the 3-gentiobiosides as the major compounds [70]. The authors also found traces of a flavone (tricin) glycoside in *T. pentandrus.* The above study permitted the authors to separate the Tribulaceae as a distinct family from Zygophyllaceae [70]. Furthermore, Hamid et al. observed that native steroidal glycosides of both *T. pentandrus* and *T. megistopterus* subsp. *pterocarpus* were very similar to each other, but that steroidal glycosides from *T. parvispinus* was remarkably different [71]. The authors elucidated the 10 chemical structures (**104**–**113**) of the related aglycones that have been reported from the genus *Tribulus* [71], meanwhile Mahalel isolated the steroidal glycoside pentandroside A (**114**) from the aerial parts of *T. pentandrus* and evaluated its antimicrobial activity [72]. Pentandroside A exhibited strong activities against *B. cereus*, *S*. *aureus, S. marcescens, E. coli*, and *P.putida*, while also exhibiting strong susceptibility against *E. coli* at all tested concentrations, the largest clearing zone being at 100 μg/disc (14 mm). The results further showed that pentandroside A was more effective than ampicillin, cephaloridine and pencillin G and similar to cortimoxazole at concentration 25 μg/disc (11 mm of diameter). Compound **114** had been previously isolated from the same plant species, along with the related analogues; pentandrosides B-G (**115**–**120**), although the isolated compounds were not previously tested [73]. Furthermore, the flavonoid glycosides; quercetin 3-*O*-glycoside (**121**), rutin or quercetin 3-*O*-rutinoside (**122**) and kaempferol 3-*O*-glycoside (**123**) were isolated from *T. terrestris* harvested in Iran [87].
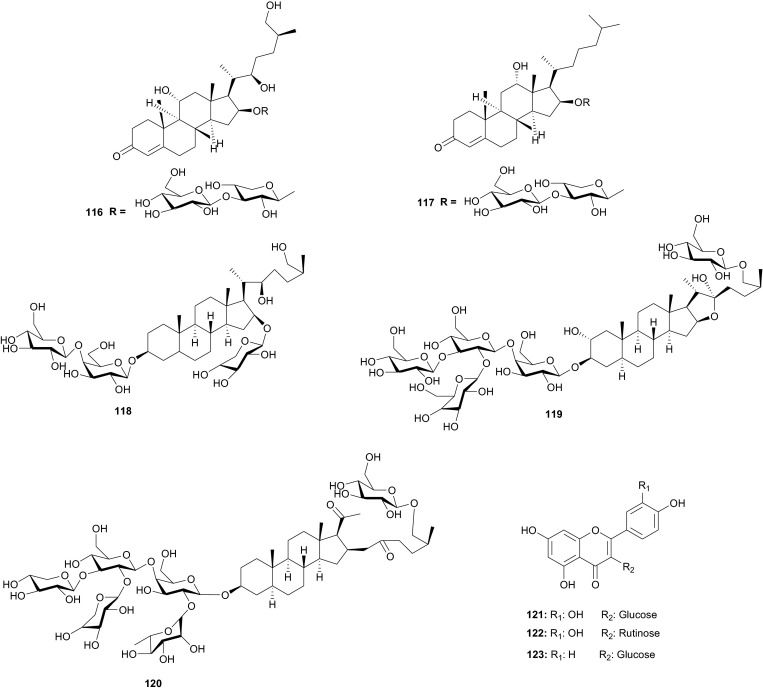


## Ulvaceae, Umbelliferae and Urticaceae

Ulvaceae contain polyunsaturated fatty acids with algicidal activities, phenolic compounds with antioxidant activities, sterols, terpenss and polyphenolic compounds [88–90]. A summary of the medicinal uses and biological activities of the compounds of the above plant families are indicated in Table 3. Marine algae (Ulvaceae) are known for their wide variety of uses as food, in industry and in medicine [91]. Additionally, their bioactive components have a broad range of pharmacological functions [88–95]. *Ulva lactuca* (commonly known as sea lettuce), for example can be used in salads and soups, ice cream, other food products, and medicine [96]. The species is also used to monitor environmental pollution, since the plant thrives in moderate levels of nutrient pollution [96]. Awad isolated the steroid 3-*O*-β-d-glucopyranosyl-stigmasta-5,25-dien (**124**), which exhibited topical anti-inflammatory activity in the mouse ear oedema assay [95]. Compound **124** as well showed antimicrobial activity against a broad range of Gram-positive and Gram-negative bacteria, fungi and yeast strains [95]. This proves that the consumption of the plant as food may be of both dietary and medicinal importance. El Ashry et al. also carried out phytochemical investigations of the same species, leading to the isolation of two new compounds; (*E*)-6-heptacosen-5-one (**125**) and (*E*)-6-octadecen-5-ol (**126**), along with four known compounds; (*Z*)-10-hexacosene (**127**), docosanoic acid (**128**), palmitic acid (**129**), and isofucosterol (**130**), (*Z*)-10-hexacosene and docosanoic acid being isolated for the first time from this species, although the compounds were not tested [97].
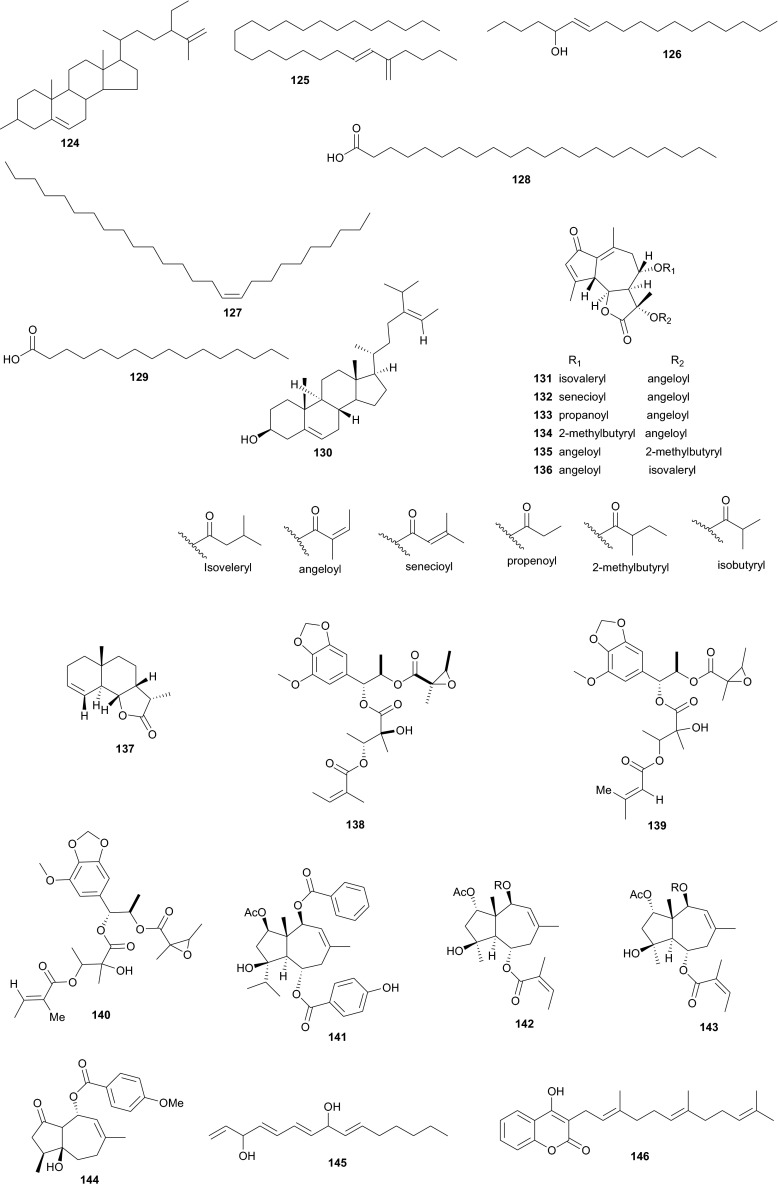

**Table 3:** Summary of ethnobotanical uses versus measured biological activities of isolated secondary metabolites from Ulvaceae, Umbelliferae and Urticaceae

Plant family	Plant name (country)	Use in traditional medicine	Part of plant studied	Isolated principle	Measured activity	Author and reference
Ulvaceae	*Ulva lactuca* (Egypt)	Used in salads and soups, ice cream, other food products, in medicine and to monitor environmental pollution [96]	Whole plant	**124**–**130**	**124** exhibited anti-inflammatory activity while 125–130 were not tested	Awad [95]; El Ashry et al. [97]
Umbelliferae	*Daucus glaber* (Egypt)	Plants of the genus *Daucus* are used as diuretics, emollient, vermifuge, carminative and against **stomach ache** [93, 94], while *Daucus carota* has edible roots [100]	Leaves and stems	**131**–**137**	**134**,** 137** and talasins A and B showed moderate cytotoxicity against P-388 leukemia cells	Sallam et al. [101]
				**138**–**140**	Cytotoxicity against P-388 murine lymphocytic leukemia cells	Sallam et al. [102]
	*Daucus carota* (carrot plant)		Plant in flowering stage	**141**–**146**	**Antimicrobial**	Ahmed et al. [103]
	*Ammi mujus* (Egypt)	Used traditionally for the treatment of skin disorders such as psoriasis and vitiligo [100–102]. Also used to treat irregular menstruation, as a diuretic, and for the treatment of leprosy, kidney stones and urinary tract infections [108]	Fruits	**147**–**158**	**Wide range** of pharmacological activities	Elgamal et al. [120]
			Aerial parts	**73**,** 159**–**161**	Anti-inflammatory activity	Selim and Ouf [129]
	*Ammi visnaga* (Algeria)	Used in Algerian folk medicine to treat vitiligo	Aerial parts	**162**–**172**	Not tested	Bencheraiet et al. [130]
	*Ferula communis* (Morocco)	The plant **intoxicates** grazing animals, although the plant has several uses in Moroccan traditional medicine [131]	Roots	**173**–**174**	**Haemorrhagic** action	Lamnaouer et al. [132]
	*Ferula hermonis* (Syria/Egypt)	Used as an aphrodisiac, and for the treatment of frigidity and **impotence**	Roots	**175**–**191**	**Antibacterial** and antioxidant	Ibraheim et al. [133]
	*Ferula lutea* (Tunisia)		Roots	**73**,** 192**–**199**	Antioxidant, anti-acetylcholinesterase and cytotoxic	Ben Salem et al. [137]
	*Ferula vesceritensis* (Algeria)	Traditionally used for the treatment of neurological disorders (tranquillizer, antihysteric), dysentery, digestive disorders, rheumatism, headache, arthritis and dizziness [138]	Aerial parts	**200**–**210**	Not tested	Oughlissi-Dehak et al. [139]
	*Carum montanum* (Algeria)	The seeds of the sister species *C. carvi* have been used in traditionally for the treatment of colic, appetite loss, anti-malarial preparations, as a vermifuge and for digestive disorders, etc.	Aerial parts	**212**–**217**	**212**–**215** showed antimicrobial properties, but 216 and 217 were not tested	Laouer et al. [142]; Benahmed et al. [143]
	*Smyrnium olusatrum* (Libya)	Used as an antiscorbutic and the young shoots are used as pot herb and edible salad	Fruits	**218**–**224**	Marginal cytotoxic activity	El-Gamal [149]
Urticaceae	*Urtica dioica* (Morocco)	Known for its antihypotensive and antidiabetic properpties [147, 148]	Roots	**225**–**230**	Vasorelaxant	Schöttner et al. [164, 165]

The Umbelliferae (apiaceous plants) are known for the presence of saponins, sesquiterpene lactones, coumarins, flavone glucosides, tanins and a broad range of volatiles. The family has been previously discussed in this review series under the Apiaceae [14], but we further highlight herein some species that were not included in the previous review and provide a more in-depth discussion of this family. Plants of the genus *Daucus* have been used by natives of some Northern African countries as diuretics, emollient, vermifuge, carminative and against stomach ache [98, 99], some of the species having edible roots, e.g. *Daucus carota* [100]. Salam et al. identified six new sesquiterpene lactone esters; daucoguaianolactones A–F (**131**–**136**), and one new sesquiterpene lactone; daucoeudesmanolactone A (**137**) from the leaves and stems of *Daucus glaber* [101], along with the known sesquiterpene lactones talasins A and B and badkhysin. Bioactivity assessment showed that compounds **134**, **137**, and talasins A and B showed moderate cytotoxicity against P-388 leukemia cells [101]. From the leaves and stems of the same species, Sallam et al. also isolated three new phenylpropanoid triesters, glaberins A–C (**138**–**140**), compounds **138** and **139** showing cytotoxicity against P-388 murine lymphocytic leukemia cells with respective IC_50_ values of 55 and 44 µg/mL [102]. Ahmed et al. rather isolated rare trisubstituted sesquiterpenes daucanes (**141**–**144**) from the sister species *D. carota*, collected from the wild [103]. The isolated compounds also included the polyacetylene falcarindiol (**145**) and the sesquiterpene coumarin ferulenol (**146**). The assessment of the antimicrobial activities of these compounds showed that the MIC values for **141** and **142** were <2 mg/mL against *Staphylococcus aureus*, *Streptomyces scabies*, *Bacillus subtilis*, *Bacillus cereus* as well as the gram negative species *Pseudomonas aeroginosa*. Meanwhile, compound **146** had an estimated MIC < 2.5 mg/mL against *S. aureus*, *S. scabies*, *B. subtilis*, *B. cereus* and *P. aeroginosa* and within the range 5 mg/mL < MIC < 4.5 mg/mL against *E. coli*, *F. oxysporum* and *A. niger*. Compounds **144** and **145** had MIC values <2.5 mg/mL against all the above tested microorganisms but showed no antibacterial effect against *F. oxysporum* and *E. coli* [103]. These results are in accordance with the use of wild carrots in traditional medicine; extract of the plant are used traditionally for the treatment of hepatic and renal insufficiency as well as for skin disorders (microbial infections) [104], while the extracts of the wild plants are also known to exhibit antioxidative and iron-chelative properties. They could also be suggested for venereal diseases, skin diseases like scabies and eczema as well as the treatment of stomach ache.

In Northern Africa, plants of the genus *Ammi* (Umbelliferae) are known to be native in Egypt and some of its species are remarkably important in traditional medicine, e.g. *A. mujus* is used traditionally for the treatment of skin disorders such as psoriasis and vitiligo (acquired leukoderma) [105–107]. The plant is also used to treat irregular menstruation, as a diuretic, and for the treatment of leprosy, kidney stones and urinary tract infections [108]. Clinical trials of the efficacy of Fructus Ammi Majoris (the dried ripe fruits of this species) and xanthotoxin for the treatment of vitiligo, psoriasis, and hypopigmentation tinea versicolor have proven positive [109–115]. Consequently, *A. mujus* is known to be one of the most important medicinal plants which has been used industrially for the isolation of its active principles in Egypt [116]. The plant has also been used in traditional medicine for xanthotoxin production [116, 117], an important drug for leukodermia treatment [118, 119]. Phytochemical investigations of the fruits of this plant by Elgamal et al. led to the identification of a new coumarin named isoarnottinin (**147**), together with two of its glucosides (**148**–**149**) [120]. Moreover, seven known coumarins; scopoletin (**150**), 8-(2**″**-acetoxy-3**″**-hydroxy-3**″**-methylbutoxy)psoralen (**151**), 8-hydroxy-5-methoxypsoralene (**152**), apiumetin (**153**), dihydroxanthyletin (**154**), lomatin (**155**) and ammirol (**156**), as well as oleanolic acid (**157**) and mannitol (**158**) were also isolated from the dried fruits [120]. The isolated compounds are known for a wide range of pharmacological activities, e.g. the glycoside of compound **147** showed high antibacterial effect against the plant pathogen *Erwinia carotovora*, with an MIC value of 100 µg/mL [121]. Moreover, this compound also exhibited significant phytotoxic activity against lettuce and modest cytotoxic activity against HeLa cell line with an IC_50_ value of 0.84 mg/mL [107]. Thus, the authors of this study concluded that the isolate isoamottinin 4′-glucoside may play a phytoalexin or allelopathic role for plants and that this compound may be a candidate for an antibacterial agent or a bioherbicide, beyond its other known activities [122]. Scopoletin is known to exhibit a broad range of pharmacological activities, including; antifungal [123], acetycholinesterase inhibition [124], insect feeding deterrent and growth inhibitory activities [125] and aldose reductase inhibitory activity [126], while the coumarin and its derivatives have also shown antitumour actvities [127, 128]. Additionally, Selim and Ouf recently isolated the coumarins 6-hydroxy-7-methoxy-4-methyl coumarin (**159**), 6-hydroxy-7-methoxy coumarin (**160**) and xanthotoxin (**161**), together with β-sitosterol (**73**) from the aerial parts of the plant [129]. Meanwhile, Bencheraiet et al. isolated the flovonoids; quercetin (**162**) rhamnetin (**163**), isorhamnetin (**164**), rhamnazin (**165**), three 3-*O*-glucosides respectively linked to; rhamnetin (**166**), isorhamnetin (**167**) and rhamnazin (**168**); one 7-*O*-glucoside of isorhamnetin (**169**), two diglycosides; 3-*O*-rutin of quercetin (**170**) and isorhamnetin (**171**), together with quercetin 7,3,3′-*O*-triglucoside (**172**) from the sister species, *A.visnaga*, collected from Algeria (used in Algerian folk medicine to treat vitiligo) [130]. The biological tests showed that compounds **159** and **160** are good anti-inflammatory agents, both compounds exhibiting appreciable inhibition of edema, particularly compound **159**, which exhibited an 87 % edema inhibition of 37.81 %, comparable with that of the standard drug indomethacin (60.50 % at 0.01 mg/100 g dose), while compound **161** exhibited mild anti-inflammatory activity [129]. On the contrary, all coumarins from *A. mujus* (**159**–**161**) were found to have no reliable antiviral activity against herpes simplex virus (HSV).
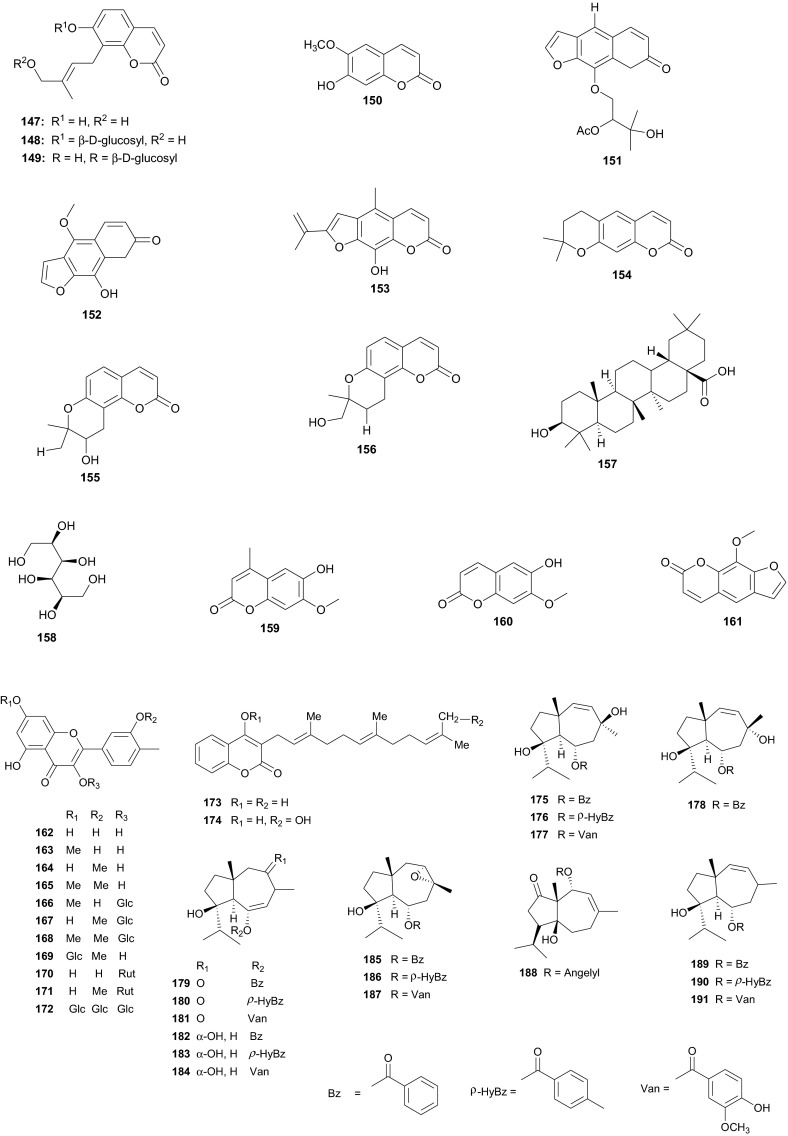

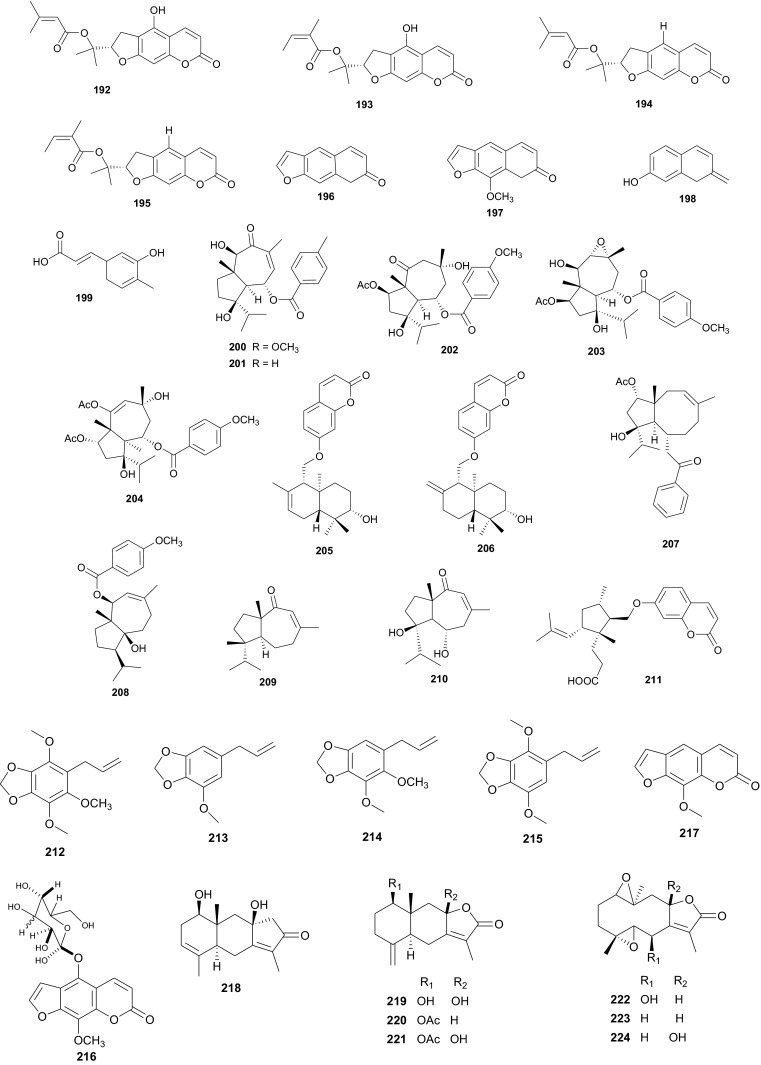


*Ferula communis* (Umbelliferae) is a perennial weed found in several Mediterranean countries, the leaves of which are known amongst Arab cattle rearers to intoxicate animals, although the plant has several uses in Moroccan traditional medicine [131]. Lamnaouer et al. have identified the toxic products of this plant to be two isoprenylated 4-hydroxycoumarins; ferulenol (**173**) and ω-hydroxyferulenol (**174**, isolated from this plant for the first time), both natural products showing haemorrhagic action [132]. The roots of the sister species, *F. hermonis*, are known to be the source of seventeen antibacterial and antioxidant daucane sesquiterpenoids (**175**–**191**) [133]. This plant has long been used in the Middle East as an aphrodisiac, and for the treatment of frigidity and impotence [134, 135]. The isolated compounds exhibited DPPH radical scavenging activities, with IC_50_ values varying from 11.5 to 183.5 µM, with teferidin (**189)**, ferutinin (**190)** and teferin (**191)** notably exhibiting the highest radical scavenging activities, comparable with that of the standard drug Ascorbic acid (IC_50_ = 12.5 µM). Geroushi et al. had previously isolated the anti-inflammatory sesquiterpenes (**189**–**191**) from the root oil of *F. hermonis* [136]. The antimicrobial activity of the isolated metabolites (**189**–**191**) was also evaluated by determination of MIC using the broth microdilution method against six bacterial strains and one fungal strain (*Pseudomonas aeruginosa* PAO1, *Escherichia coli*, *Bacillus subtilis* ATCC6633, *Mycobacterium bovis* BCG Pasteur, *Mycobacterium tuberculosis* H37Rv, *Staphylococcus aureus* ATCC6538 and *Candida albicans* SC5314). Again, compounds **189**, **190** and **191** demonstrated potent activity against the Gram +ve (*S. aureus*, *B. subtilis*), as well as the *Mycobacterium* strains *M. bovis* BCG and *M. tuberculosis* H37Rv. None of the isolated compounds exhibited a significant antifungal activity [133–135]. The roots of the sister Tunisian species *F. lutea* have rather been characterized by antioxidant, anti-acetylcholinesterase and cytotoxic dihydrofuranocoumarins; (−)-5-hydroxyprantschimgin (**192**) and (−)-5-hydroxydeltoin (**193**) [137]. These compounds were isolated along with eight known compounds; (−)-prantschimgin (**194**), (−)-deltoin (**195**), psoralen (**196**), xanthotoxin (**197**), umbelliferone (**198**), caffeic acid (**199**), β-sitosterol (**73**) and stigmasterol. Meanwhile the aerial parts of the Algerian species, *F. vesceritensis*, traditionally used for the treatment of neurological disorders (tranquillizer, antihysteric), dysentery, digestive disorders, rheumatism, headache, arthritis and dizziness [138], has been characterized by the presence of sesquiterpenes and sesquiterpene coumarins [139]. Oughlissi-Dehak et al. analysed the chemical composition of the dichloromethane extract of aerial parts of the plant and found them to be composed of five new sesquiterpenes (**200**–**204**), together with six related compounds (**205**–**210**) identified, respectively as; feselol (**205**), farnesiferol A (**206**), 2-acetyl-jaechkeanadiol-6-anisate (**207**), lasidiol-10-anisate (**208**), 10-oxojaesckeanadiol-6-anisate (**209**) and lapidol (**210**) [139]. Moreover, ferulsinaic acid (**211**) is a sesquiterpene coumarin with a rare carbon skeleton, known to be the taxonomic marker of the genus *Ferula* [140].

Plants of the genus *Carum* (Umbelliferae) have a wide range of industrial and pharmacological uses, e.g. *C. carvi* (also known as Caraway) has been used in Europe for flavouring bread, sauerkraut, candies, meat products, sauces, cheese and alcoholic liqueurs [141, 142]. Moreover, the seeds of this plant have been used traditionally for the treatment of colic, appetite loss, anti-malarial preparations, as a vermifuge and for digestive disorders, etc. [142]. Laouer et al. have investigated the essential oil of the aerial parts (leaves and flowers) of the sister species, *C.montanum* (a plant commonly grazed by livestock), harvested from the Megress Mountain in Algeria, although no medicinal applications are known for the Algerian species locally [142]. This study led to the isolation of phenylpropanoids with antimicrobial properties [142]. These include; nothoapiole (**212**), myristicin (**213**), apiole (**214**) and dillapiole (**215**) [142]. Meanwhile, Benahmed et al. had previously isolated the new furanocoumarin glucoside, xanthotoxin-5-*O*-β-d-glucoside named carumoside (**216**), together with the known coumarin xanthotoxin (**217**), from aerial parts of the Algerian species, but did not test the isolated compounds [143]. It should be noted that although coumarins are common in apiaceous plants (particularly within the genera *Apium*, *Ammi* and *Magydaris*) [144–148], the presence of the coumarin xanthotoxin (**217**) in both species (*C. carvi* [146] and *C. montanum* [143]) and the concurrent absence of its 5-glucosides in *C. carvi* may be an indication that the biosynthesis of 5-glucosides of furanocoumarins is a distinctive characteristic of the Algerian species, *C. montanum* [143].
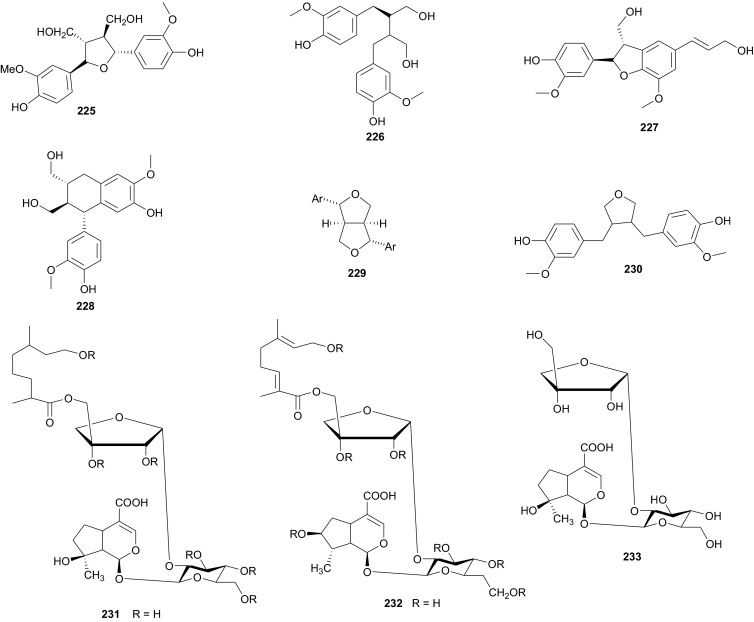


*Smyrnium olusatrum* (Umbelliferae) is a well known medicinal plant, which is locally cultivated in Libya where the young shoots are used as pot herb and edible salad in some areas of the country [149]. The plant is also reported to be used as an antiscorbutic in Palestine [150]. El-Gamal examined the fruits of this plant and isolated three sesquiterpene lactones, namely; 1β,8β-dihydroxy eudesman 3,7(11)-dien 8α,12 olide (**218**), 1β,8β-dihydroxy eudesman 4(15),7(11)-dien 8α,l2 olide (**219**) and 1β,10α;4α,5β-diepoxy 6-β-hydroxy-glechoman-8α,12 olide (**220**), together with four related known sesquiterpenes (**221**–**224**) [149]. Although previous in vitro cytotoxicity assays had showed that the CH_2_Cl_2_ extract of the fruits of the plant exhibited significant cytotoxicity (IC_50_ = 9.0 µg/mL) against P-388 mouse lymphoma cells [151, 152], the study by El-Gamal rather showed that the isolated compounds (**218**, **219** and **221**–**224**) only showed marginal activity, with respective IC_50_ values of 60, 65, 42, 58, 94 and 88 µg/mL [149].

From the Urticaceae, *Urtica dioica* (or nettle) is widely used as a food supplement (with salad) in the East of Morocco [153]. The plant is also known in Eastern Moroccan traditional medicine for its antihypotensive and antidiabetic properties [154, 155], meanwhile its extracts have displayed hypoglycemic [153, 156], antioxidant, antimicrobial, antiulcer and analgesic [157], antiproliferative [158, 159], antirheumatic [160] and hepatoprotective [161] effects. It has also been demonstrated that a preparation containing several medicinal plants, including *U. dioica* exhibited antidiabetic activity [162] and could also be used to treat allergic rhinitis [163]. Bnouham et al. investigated the hypoglycemic effect of aqueous extract of *U. dioica*, harvested from Eastern Morocco, on hyperglycemia induced by oral glucose tolerance test (OGTT) and on alloxan-induced diabetic rats, with the view of determining one of the probable mechanisms of nettle’s antihyperglycemic effect [149]. Their results showed that *U. dioica* had a significant antihyperglycemic effect which may be partly as a result of the reduction of intestinal glucose absorption, thus suggesting that the plant is recommendable as a food supplement for the management of diabetes. Schöttner et al. have isolated the following ligans; (+)-neoolivil (**225**), (−)-secoisolariciresinol (**226**), dehydrodiconiferyl alcohol (**227**), isolariciresinol (**228**), pinoresinol (**229**) and 3,4-divanillyltetrahydrofuran (**230**) from the root polar extracts of the plant [164, 165]. The compounds were tested for their affinity to human sex hormone binding globulin (SHBG) and results showed that all lignans except (−)-pinoresinol developed a binding affinity to SHBG in the in vitro assay, with the affinity of (−)-3,4-divanillyltetrahydrofuran proving to be significantly high, when compared with those of the other isolates [164]. These results could suggest the further examination of these compounds (**225**–**228** and **230**) and plant lignans in general for the development of drugs against benign prostatic hyperplasia (BPH).

## Verbenaceae and Vitaceae

Verbenaceae contain triterpenoids, flavonoids, chromomoric acid derivatives, quinones, apocarotenoids and lignans [166], while Vitaceae are known to be rich in flavonoids, terpenes, organic acids, vitamins, carbohydrats, lipids and enzymes [167]. A summary of the medicinal uses and biological activities of the compounds of the plant families above are indicated in Table 4. Flowering plants of the genus *Clerodendrum* (formerly Verbenaceae, now Lamiaceae) are currently classified in the subfamily Ajugoideae [168, 169]. The species have diverse uses in folk medicine, e.g. the aqueous leaf extract of *C. glandulosum* is traditionally used by people of North-East India to alleviate symptoms of diabetes, obesity and hypertension, while the plant is used to prepare a staple diet among the Zomi tribes (North East India) [170]. The leaf juice of the sister species *C. inerme* (commonly used as a hedge plant) is reportedly used as a remedy to bring down fever [171]. Phytochemical investigation of the leaf extracts of *C. inerme* from Giza in Egypt led to the identification of five novel complex iridoid glycosides; inerminosides A and B (**231** and **232**) [172], as well as inerminosides A1, C and D (**233**–**235**) [173], although the compounds were not tested. Therefore, the validation of the ethnobotanical uses of *Clerodendrum* species from Northern Africa is still under investigation.
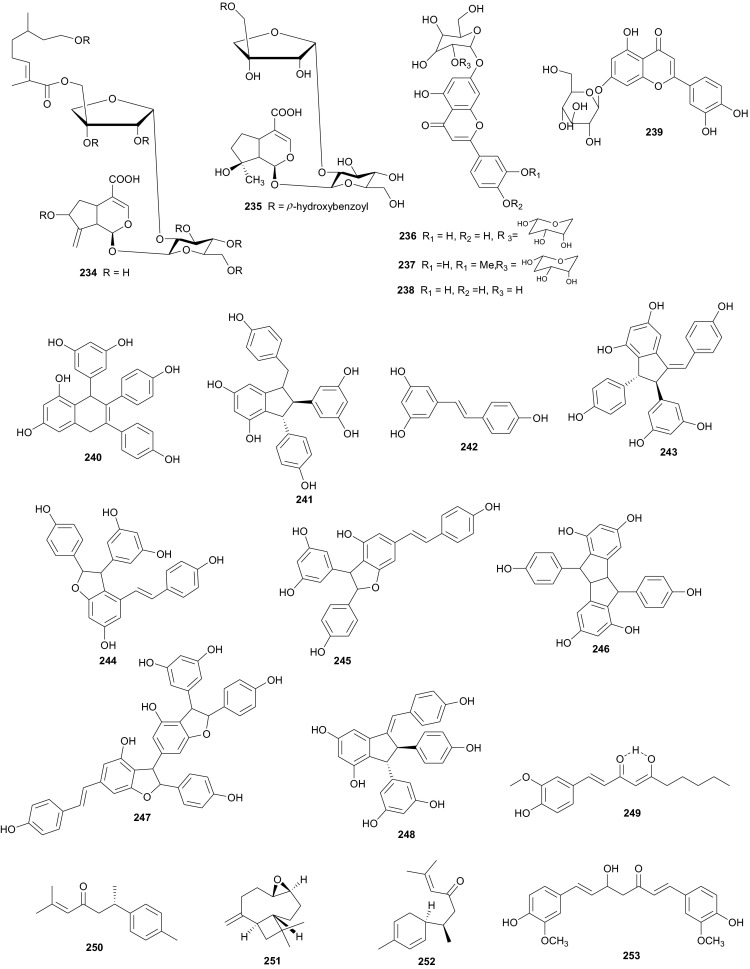

**Table 4:** Summary of ethnobotanical uses versus measured biological activities of isolated secondary metabolites from Verbenaceae and Vitaceae

Plant family	Plant name (country)	Use in traditional medicine	Part of plant studied	Isolated principle	Measured activity	Author and reference
Verbenaceae	*Clerodendrum inerme* (Egypt)	The leaf juice is used as a remedy to bring down fever [171]	Leaves	**231**–**235**	Not tested	Caliş et al. [172, 173]
	*Verbena officinalis* and *V. supina* (Egypt)	*Verbena officinalis* is used as tea or liqueur for the treatment of infections and fever [174]	Aerial parts	**236**–**239**	Not tested	Kawashty and El-Garf [176]
Vitaceae	*Cyphostemma crotalarioides* (Sudan)	The roots are used to prepare pain-killers and for stomach troubles [178]	Roots	**240**–**248**	Cancer chemopreventive, antifungal and antibacterial activities	Ducrot et al. [177]

*Verbena officinalis* (Verbenaceae) is a herb that has been used in traditional Austrian medicine (as tea or liqueur) for the treatment of infections and fever [174]. Vebena, a common food supplement derived from this plant, is used for sore throats and respiratory tract diseases such as asthma and whooping cough, and for heart conditions such as chest pain (angina) and fluid retention due to heart failure [175]. An investigation of *V. officinalis* and the sister species *V. supina* collected in Egypt, led to the identification of several flavonoid glycosides which permitted to chemically distinguish between the two species [176, 177]. Although the compounds were not tested, the favonoid profiles of the species indicated that luteolin-7-neohesperidoside (**236**) and diosmetin-7-neohesperidoside (**237**) are major components in *V. officinalis* while the major component of *V. supina* was tuteolin-7-glucoside (**238**). Both species contained luteolin-7-galactoside (**239**) and diosmetin-7-galactoside, together with apigenin-7-glucoside and chrysoeriol-7-galactoside in minor quantities [176, 177].

*Cyphostemma crotalarioides* (Vitaceae) is a plant known in Sudan for its applications in pest management programs [176, 177]. The roots are also known to be used in traditional preparations as pain-killers and for stomach troubles [178]. To confirm the use of the plant to treat stomach troubles, Ducrot et al. have successfully isolated two new antifungal oligostilbenes; cyphostemmins A and B (**240** and **241**) [176, 177], along with resveratrol (**242**) and some of its known natural oligomers; namely parthenocissin A (**243**), ε-viniferin (**244**), gnetin C (**245**), pallidol (**246**), gnetin E (**247**) and ampelopsin D (**248**) [179], from the roots of the plant species collected from Sudan. Compound **242** and it’s analogues exhibited cancer chemopreventive, antifungal and antibacterial activities [179–189].

## Zingiberaceae and Zygophyllaceae

A summary of the medicinal uses and biological activities of the compounds of the above plant families are indicated in Table 5. The Zingiberaceae are plants possessing rhizomes, including species like ginger and cardamom, which are commonly used as spices [190]. This family is known for their labdane diterpenoids. The rhizome powder of Turmeric (*Curcuma longa*, Zingiberaceae), for example, is often added to various food preparations to preserve their freshness and impart a characteristic flavour. In India, traditional claims hold that Turmeric powder is effective against biliary disorders, anorexia, coryza, cough, diabetic wounds, hepatic disorder, rheumatism, and sinusitis [191]. Demerdash et al. have isolated four compounds, namely; 1-dehydrogingerdione (**249**), *ar*-turmerone (**250**), (−)-caryophyllene oxide (**251**) and α-turmerone (**252**) from a commercial powdered sample purchased from a local market in Mansoura, Egypt [192]. Although the authors did not report any biological tests, this plant together with the sister species *C. comosa* are known to be rich sources of diarylheptanoids (e.g. Curcumin, **253**) [193, 194], which are known for their antioxidant [195], antiviral [196], melanogenesis-inhibitory and cytotoxic [197–200], anti-Leishmanial [201] and anti-Trypanosomal [202] activities, just to mention these ones.

**Table 5:** Summary of ethnobotanical uses *versus* measured biological activities of isolated secondary metabolites from Zingiberaceae and Zygophyllaceae

Plant family	Plant name (country)	Use in traditional medicine	Part of plant studied	Isolated principle	Measured activity	Author and reference
Zingiberaceae	*Curcuma longa* (Egypt)	Added to various food preparations to preserve their freshness and impart a characteristic flavour	Powdered root	**249**–**252**	Not tested	Demerdash et al. [192]
Zygophyllaceae	*Tribulus parvispinus* (Egypt)	Species of the genus *Tribulus* have a broad range of uses in folk medicine, e.g. the fruits of *T. alatus* for the treatment of urinary disorders and cough [197, 198], while *T. terrestris* has been used for the treatment of impotence, rheumatism, edema, hypertension and kidney stones [199–201]	Aerial parts	**254**–**259**	Cytotoxic activity	Perrone et al. [203]
	*Tribulus alatus* (Egypt)		Aerial parts	**260**–**265**	Not tested	Temraz et al. [204]
	*Tribulus pentandrus* (Egypt)		Aerial parts	**266**–**272**	Not tested	Hamed et al. [73]
	*Tribulus terrestris* (Egypt)		Aerial parts	**273**–**280**	Antioxidant activity	Hammoda et al. [205]
	*Paganum harmala* (Egypt)	The seeds are used for cancer treatment [243]	Seeds	**281**–**284**	Antimicrobial	Nenaah [241]

The Zygophyllaceae are known for their flavonoid content. The family has been briefly discussed in this series previously under Balanitaceae, but the report was focused on the genus *Balanites* [14], thereby ignoring the previously investigated genera *Tribulus*, *Fagonia*, *Zygophylum* and *Paganum*). In the genus *Tribulus*, the Egyptian species *T. parvispinus* [203], *T. alatus* [204], *T. pentandrus* [73], and *T. terrestrris* [205] have been mainly investigated. With a wide range of uses in folk medicine, e.g. the fruits of *T. alatus* are used in Pakistan for the treatment of urinary disorders and cough [206, 207]; *T. terrestris* has been used for the treatment of impotence, rheumatism, edema, hypertension and kidney stones [208–210], since the extract of the plant possesses aphrodisiac properties probably due to androgen increasing properties [211, 212]; the *Tribulus* species are known to be rich in furostane- and spirostane-type steroidal saponins which exhibit a broad range of pharmacological functions, e.g. cytotoxic [213], antiproliferative [214], and antimicrobial effects [215]. The most important isolated compounds from the Northern African species include; furostanol saponins (**254**–**259**) from *T. parvispinus*, exhibiting cytotoxic activities against U937, MCF7, and HepG2 cells, with compounds **255** and **258**, having IC_50_ values of 0.5 and 0.1 µM, respectively against U937 cells [203]. The untested steroidal glycosides (**260**–**265**) were isolated from the aerial parts of *T. alatus* [204], while seven new steroidal glycosides; pentandrosides A-G (**266**–**272**) were isolated from the aerial parts of *T. pentandrus*, with **272** being an unusual acyloxypregnane aglycone probably derived from the degradation of a furostan skeleton [73]. From the aerial parts of *T. terrestris*, Hammoda et al. isolated two oligosaccharides (**273** and **274**) and a stereoisomer of di-*p*-coumaroylquinic acid (**275**), together with five known compounds (**276**–**280**) [205]. The isolates exhibited antioxidant activities, with compound **276** (4,5-di-*p*-*trans*-coumaroylquinic acid), showing significantly stronger DPPH free radical scavenging activity than the total ethyl acetate fraction, while the IC_50_ values of the geometric isomers **275** and **276** being comparable with that of the reference compound (Ascorbic acid) [205].
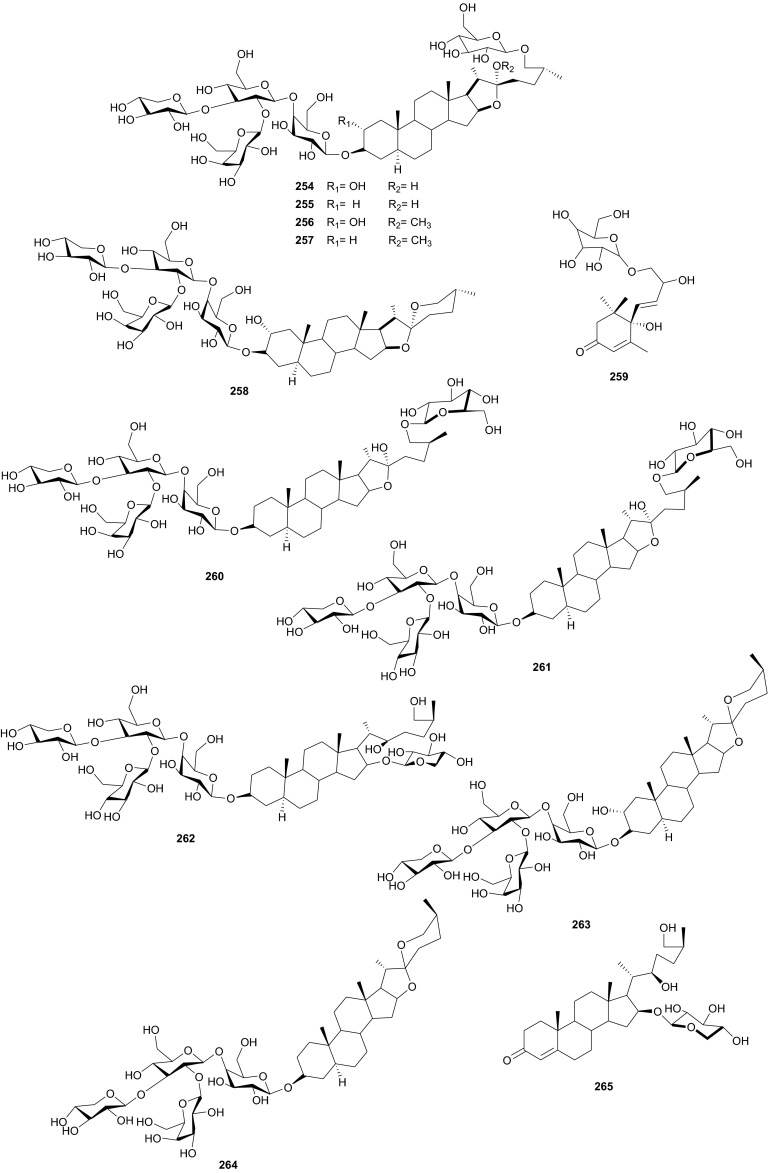

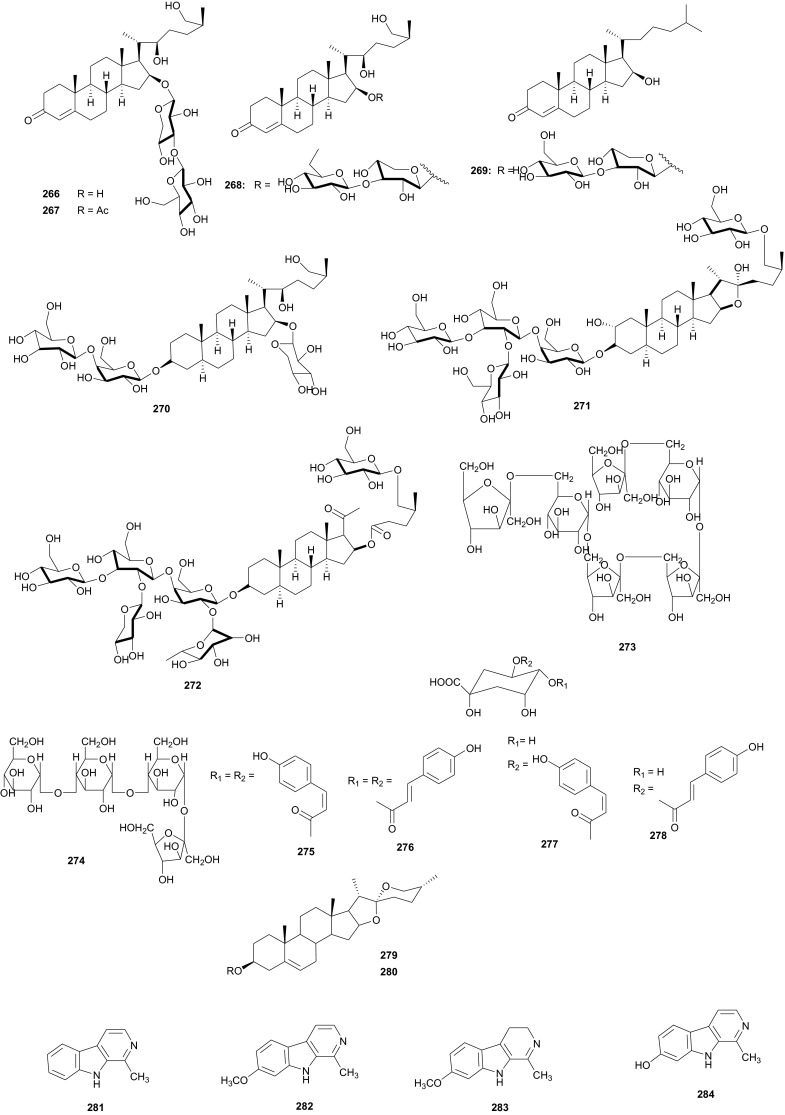


The *Fagonia* [216–229] and *Zygophylum* [230–239] genera have been extensively explored and reviewed. Therefore a complete discussion is beyond the scope of this article. Within the genus *Paganum* the only species investigated was *P. harmala* [240–242], a plant whose seeds are used for cancer treatment [243], and which are reported to contain between 2 and 6 % biologically active alkaloids, mainly β-carbolines, e.g. harman (**281**), harmine (**282**), harmaline (**283**) and harmalol (**284**) [241, 244–246]. The methanol extract of seeds of the plant harvested in Morocco significantly reduced the proliferation of three tumoral cell-lines; UCP-Med and Med-mek carcinoma, and UCP-Med sarcoma at concentrations of 20–120 µg/mL during the first 24 h of contact [240]. The alkaloids (**281**–**284**) were derived from the species harvested in Egypt [241], together with four untested flavonoid glycosides [242]. The isolated alkaloids also exhibited antimicrobial activties against a wide range of organisms, including; *Escherichia coli*, *Proteus vulgaris*, *Staphyllococcus aureus*, *Bacillus subitilis*, *Asperagillus niger* and *Candida albicans* [241].

## Discussion and Conclusions

This review paper is not an attempt to present an exhaustive overview of compounds from Northern African plant species and their biological activities, the reason being that such an attempt is not feasible in a journal article. The main goal of this review series has been to establish a baseline for further investigations of North African flora. Data on plant sources, geographical collection sites and chemical structures of pure compounds have been retrieved from literature sources (mainly from the major international journals on natural products and some available PhD theses, spanning the period 1959–2015). From Taccaceae to Zygophyllaceae, we have discussed 284 metabolites which have been isolated from 34 plant species belonging to 11 Northern African plant families. From Tables 1 to 5, it appears as though the correlation between the application of plant materials (e.g. in traditional medicines) and the biological activities of the isolated plant metabolites is relatively weak (with the exception of a few correlations marked in bold), when compared with our previous reviews from Central Africa [247, 248], West Africa [249–252], Southern Africa [253, 254] and the previously discussed families from the region [14, 15]. One could easily explain off this weak correlation by the fact that traditional methods like boiling, the use of total extracts as mixtures, steam baths, etc. do not match the typical chemical extraction methods employed in a NP laboratory. The survey however portrays the importance of traditional medicine in drug discovery programs. Previous surveys have also demonstrated that NPs from African flora have shown a great diversity of chemical classes with a broad range of biological activities; including >500 compounds with anti-malarial activties [255, 256], ~100 compounds with potential anti-mycobacterial activities [257], >400 compounds with potential anticancer activities [258, 259] and ~100 compounds with anti-trypanosomal activities [260, 261]. This leaves us with a huge repository for drug discovery. It should be noted that the tested compounds only represent a small proportion of NPs from the region which have been isolated. The chemical scaffolds from the untested NPs still remain to be explored. From this data and the previously presented reviews, we are currently building a searchable database for compounds isolated from Northern African flora, in order to assist in drug discovery programs on the continent of Africa.

## Electronic supplementary material

Supplementary material 1 (DOC 33 kb)
