# Supplementary material for: The Chemistry and Biological Activities of Natural Products from Northern African Plant Families: From Taccaceae to Zygophyllaceae
Source: Nat Prod Bioprospect. 2016 Mar 1;6(2):63–96. doi: 10.1007/s13659-016-0091-9 (PMC4805656; doi:10.1007/s13659-016-0091-9)
Supplement: Supplementary file 1 — Supplementary material 1 (DOC 33 kb) [file 13659_2016_91_MOESM1_ESM.doc]

The chemistry and biological activities of natural products from Northern African plant families: From Taccaceae to Zygophyllaceae

Fidele Ntie-Kang, *^a,b*^* Leonel E. Njume,*^c^* Yvette I. Malange,*^b^* Stefan Günther,*^d^* Wolfgang Sippl*^a^* and Joseph N. Yong*^b^*

*^a^* Department of Pharmaceutical Chemistry, Martin-Luther University of Halle-Wittenberg, Wolfgang-Langenbeck-Str. 4, 06120 Halle (Saale), Germany; Tel.: +49 345 5525 043; E-mail: fidele.ntie-kang@pharmazie.uni-halle.de

*^b^* Department of Chemistry, Faculty of Science, University of Buea, P.O. Box 63, Buea, Cameroon; Tel.: +237 677915473; E-mail: ntiekfidele@gmail.com OR fidele.ntie-kang@ubuea.cm OR joseph.yong@ubuea.cm.

*^c^* Chemical and Bioactivity Information Centre, Department of Chemistry, Faculty of Science, University of Buea, P.O. Box 63, Buea, Cameroon.

*^d^* Institute of Pharmaceutical Sciences, Research Group Pharmaceutical Bioinformatics, Albert-Ludwigs-Universität Freiburg, Hermann-Herder-Strasse 9, 79104 Freiburg, Germany

List of Journals Consulted in Constructing The Northern African Compound Database

| Journal type | List |
| --- | --- |
| International | *Acta Crystallographica*, *African Journal of Health Sciences*, *Asian Journal of Traditional Medicine*, *Arabian Journal of Chemistry*, *Crystallographica*, *Asia-Pacific Journal of Tropical Medicine*, *Biochemical Systematics and Ecology*, *Bioorganic and Medicinal Chemistry*, *Bioorganic and Medicinal Chemistry Letters*, *Bioscience Biotechnology and Biochemistry*, *Biological and Pharmaceutical Bulletin*, *BMC Complementary and Alternative Medicine*, *BMC Research Notes*, *Boletín Latinoamericano y del Caribe de Plantas Medicinales y Aromáticas*, *Bulletin of the Chemical Society of Ethiopia*, *Carbohydrate Research*, *Chemistry and Biodiversity*, *Chemical and Pharmaceutical Bulletin*, *Chemistry of Natural Compounds*, *Chinese Chemical Letters*, *Evidence Based Complementary and Alternative Medicin*e, *Fitoterapia*, *Helvetica Chimica Acta*, *Inflammopharmacology*, *Journal of Natural Products*, *Journal of Asian Natural Products Research*, *Journal of Ethnopharmacology*, *Journal of Medicinal Plants Research*, *Journal of the American Chemical Society*, *Journal of the American Oil Chemistry Society*, *Journal of Organic Chemistry*, *Journal of Pharmacognosy and Phytotherapy*, *Malaria Journal*, *Molecules*, *Natural Product Communications*, *Natural Product Letters*, *Natural Product Research*, *Natural Product Science*, *Organic and Medicinal Chemistry Letters*, *Pakistani Journal of Medical Science*, *Parasitology Research*, *Pharmaceutical Biology*, *Pharmacologia*, *Pharmacologyonline*, *Pharmazie*, *Phytochemical Analysis*, *Phytochemistry*, *Phytochemistry Letters*, *Phytopharmacology*, *Pharmaceutical Biology*, *Phytotherapy Research*, *Phytomedicine*, *Phytomedicine Reserach*, *Planta Medica*, *Planta Medica Letters*, *PLoS One*, *Pure and Applied Chemistry*, *Rasayan Journal of Chemistry*, *Records of Natural Products*, *Research Journal in Phytochemistry*, *Research Journal in Medicinal Plants*, *RSC Advances*, *South African Journal of Botany*, *Tetrahedron*, *Tetrahedron Letters*  and *Zeitschrift für Naturforschung*. |
